# Protective Effect Against Acute Experimental Toxoplasmosis Conferred by Intranasal Immunisation with *Toxoplasma gondii* Membrane Proteins Plus CpG Adjuvant

**DOI:** 10.3390/vaccines14060539

**Published:** 2026-06-17

**Authors:** Carina Brito, Daniela Teixeira, Paula Goulart, Beatriz Rodrigues, Nuno Carvalho, Manuel Vilanova, Alexandra Correia, Margarida Borges

**Affiliations:** 1 Associate Laboratory i4HB-Institute for Health and Bioeconomy, Faculty of Pharmacy, University of Porto, 4050-313 Porto, Portugal; 2 UCIBIO-Applied Molecular Biosciences Unit, Laboratory of Biochemistry, Department of Biological Sciences, Faculty of Pharmacy, University of Porto, 4050-313 Porto, Portugal; 3 Department of Biological Sciences, Faculty of Pharmacy, University of Porto, Rua de Jorge Viterbo Ferreira, 228, 4050-313 Porto, Portugal; 4 Departamento de Imuno-Fisiologia e Farmacologia, Instituto de Ciências Biomédicas Abel Salazar, University of Porto, Rua Jorge Viterbo Ferreira, 4050-313 Porto, Portugal; 5 I3S Instituto de Investigação e Inovação em Saúde da Universidade do Porto, University of Porto, Rua Alfredo Allen, 4200-135 Porto, Portugal

**Keywords:** toxoplasmosis, intranasal vaccine, systemic sustained mucosal immunity, nasal delivery

## Abstract

Background: Toxoplasmosis is a prevalent zoonotic disease worldwide, affecting approximately one-third of the global human population. Primary infection with *Toxoplasma gondii* during pregnancy can induce miscarriage or congenital infection, leading to irreversible damage to the foetus. Moreover, reactivation of *T. gondii* infection in immunosuppressed individuals can result in fatal outcomes. No vaccine exists to prevent human disease caused by this parasite. Thus, a vaccine that could induce complete and lasting protection against human toxoplasmosis is an unmet need. Method: In this work, BALB/cByJ mice were intranasally immunised with a subunit vaccine consisting of *T. gondii* membrane proteins (TGMP) from the *T. gondii* Me49 strain plus CpG-oligodeoxynucleotide adjuvant (CpG). Antibody responses were analysed by ELISA, while T-cell responses were evaluated by flow cytometry. The immunogenic proteins present in TGMP were identified by mass spectrometry, and parasite burden was quantified by qPCR. Result: The results showed raised TGMP-specific serum IgG and intestinal IgA antibody levels, and parasite-specific IFN-γ-producing CD4^+^ and CD8^+^ memory T cells. Dense granule proteins (GRA) 2 and 7, surface antigen (SAG)-related sequences 25, 29B, and 34A, microneme protein (MIC) 10, toxofilin, nascent polypeptide-associated complex (NAC) domain-containing protein, and NAC subunit beta were identified as immunogenic proteins. Mice immunised with TGMP+CpG were challenged with *T. gondii* tachyzoites and showed a significant reduction in the parasitic burden in the peritoneal exudate, spleen, and lungs, compared to mice sham-immunised with CpG alone. Conclusions: Altogether, these results indicate that mucosal immunisation with TGMP plus CpG adjuvant is worth exploring as a vaccination approach to prevent toxoplasmosis.

## 1. Introduction

Toxoplasmosis is a disease caused by *Toxoplasma gondii*, an obligate intracellular protozoan parasite that significantly impacts human health, affecting approximately two billion people worldwide [1]. Its ubiquity varies significantly across countries, with moderate prevalence in central and southern Europe and high prevalence in Latin America and tropical Africa [2]. *T. gondii* infection severely affects immunosuppressed individuals, causing clinical manifestations, such as cerebral encephalitis, that may result in fatal outcomes [3]. Moreover, in primoinfected pregnant women, toxoplasmosis may lead to devastating consequences for the foetus. Depending on the pregnancy trimester when infection occurs, abortion or congenital toxoplasmosis (ocular disease, mental retardation, hydrocephaly) may occur [4]. Currently, the gold standard treatment for acute toxoplasmosis relies on a combination of pyrimethamine and sulfadiazine, despite its toxic effects and the need for a long-duration treatment [5]. However, it is only effective during the acute phase of infection and ineffective against latent toxoplasmosis, which can potentially and severely reactivate in immunosuppressed individuals.

Vaccination is considered an effective and adequate approach to prevent toxoplasmosis. However, no vaccine is currently available to prevent toxoplasmosis in humans, nor is one being tested in clinical trials [3]. Over the last 20 years, various experimental approaches have been used for vaccine development, including subunit vaccines targeting single or combined parasite proteins involved in host cell invasion and parasite persistence. Examples include surface antigens (SAG), rhoptry antigens (ROP), microneme antigens (MIC), and dense granule antigens (GRA), all of which are crucial for parasite-induced pathogenesis [6,7]. Despite ongoing efforts, an effective *T. gondii* vaccine for human use remains unavailable, with several studies showing variation in vaccine efficacy. However, a consensus emerged that vaccines containing multiple antigens are more effective [8]. Almost all experimental vaccines developed so far have been evaluated in mouse models, and it is not always possible to extrapolate experimental results to humans or cats. However, mouse models remain essential and offer numerous advantages, serving as a valuable tool in vaccine development, particularly for understanding the immune response and identifying potential issues before progressing to human trials [9]. The intranasal (i.n.) route enhances antigen delivery to target immune cells and induces both systemic and mucosal immunity, which are essential for protection against *T. gondii* infection [10]. Indeed, i.n. vaccination has been shown to induce the production of secretory IgA in the intestinal mucosa, the primary gateway for the entry of *T. gondii* [10]. Our strategy was based on a previously developed i.n. immunisation approach that proved highly effective in inducing long-term protection against infection caused by *Neospora caninum*, a protozoan parasite closely related to *T. gondii* [11].

In this study, an i.n. vaccine formulation composed of *T. gondii* membrane proteins (TGMP) plus a CpG adjuvant (TGMP+CpG) was developed by using key immunogenic proteins identified by immunoproteomics. Immunisation with TGMP+CpG formulation induced TGMP-specific B and T cell responses and was associated with partial host protection against acute toxoplasmosis.

## 2. Materials and Methods

### 2.1. Animals

Seven-week-old female BALB\c/cByJ (BALB/c) mice (20–25 g) were purchased from Charles River Laboratories (Saint-Germain-Nuelles, France) and kept in the animal facilities of the Institute of Biomedical Sciences Abel Salazar (ICBAS, Porto, Portugal). All animal procedures were conducted in strict compliance with Directive 2010/63/EU of the European Parliament and Council on the protection of animals used for scientific purposes (ETS 123), as well as the corresponding Portuguese legislation (Decree-Law No. 113/2013). Experimental protocols were reviewed and approved by the Institutional Animal Welfare Body (ORBEA) of ICBAS (approval no. 315/2019/ORBEA) and by the national competent authority, Direção-Geral de Alimentação e Veterinária (approval dated 2 December 2019). The study design and reporting adhered to the ARRIVE guidelines (https://arriveguidelines.org). Animals were euthanized using isoflurane overdose administered via inhalation (approximately 1 mL isoflurane applied to cotton within a sealed 50 mL chamber), followed by cervical dislocation to confirm death.

BALB/c mice were selected for this study because they mount robust and well-characterised Th1/Th2 immune responses, making them an excellent model for evaluating vaccine-induced protection against both acute and chronic toxoplasmosis [12]. Although they are relatively resistant to lethal acute infection compared to other strains, this allows for a clear assessment of immune-mediated protection, including antibody production and cellular immunity, without confounding mortality effects.

### 2.2. Cell Culture

Vero cells (ATCC, CCL-81) were used as host cells to maintain and produce *T. gondii* tachyzoites. Cells were cultured in minimal essential medium containing Earle’s salts (MEM), supplemented with 10% inactivated foetal bovine serum (FBS), 100 U/mL penicillin, 0.1 mg/mL streptomycin, and 1 mM sodium pyruvate (all from Life Technologies Europe, Bleiswijk, The Netherlands). Vero cells were cultured and incubated at 37 °C in a humidified 5% CO_2_ atmosphere. For cell collection, the supplemented MEM medium was removed, and the cells were washed with phosphate-buffered saline solution (PBS). Then, a trypsin-EDTA solution (0.25% trypsin/1 mM EDTA; Thermo-Fisher, Waltham, MA, USA) was added, and the cells were incubated at 37 °C for 5 min until they detached from the flask. Supplemented MEM medium was added to inactivate trypsin, and the cell suspension was homogenised and centrifuged at 200 *g* for 6 min at 4 °C. Subsequently, the supernatant was discarded, and the cells were resuspended in growth medium and subcultured into new flasks.

### 2.3. Parasites

The ME49 strain of *T. gondii*, used in this study, was kindly provided by Dr Marcus Meissner (Parasitology Department of the Faculty of Medicine, Heidelberg University, Germany). Tachyzoites were obtained by in vitro infection of Vero cells using an optimised protocol to ensure a consistent source of parasites with minimal host cell contamination [11]. To maintain parasite culture in vitro, semi-confluent Vero cell cultures (7 × 10^6^ cells) were inoculated with tachyzoites at a multiplicity of infection (MOI) of 2 (14 × 10^6^ parasites/flask). For parasite isolation, infected Vero cells containing intracellular tachyzoites (rosettes) were scraped using a cell scraper and centrifuged at 1500 *g* for 15 min at 4 °C. The pellet was resuspended in PBS, mechanically disrupted by repeated passage through a 25-gauge needle, and adjusted to a final volume of 40 mL with PBS before centrifugation. This washing step was repeated twice. The final pellet was resuspended in PBS and purified using a PD-10 desalting column (GE, Healthcare, Freiburg, Germany) to remove host cell debris. Parasite concentration was determined using a Neubauer chamber and 0.4% Trypan Blue (Sigma-Aldrich, St. Louis, MO, USA). The cell suspension was then used to prepare the parasite inoculum for in vivo infection experiments. The remaining suspension was centrifuged at 1500 *g*, and the parasite pellets were stored at −80 °C to prepare TGMP, or the whole *T. gondii* antigen (TgS) extract.

### 2.4. Preparation of TGMP and TgS Extracts

After isolation, *T. gondii* tachyzoites were processed into hydrophilic proteins (TgHyd), hydrophobic membrane proteins (TGMP) or TgS. For phase separation, parasite pellets were resuspended in 0.75% Triton X-114 (Sigma-Aldrich, St. Louis, MO, USA) in PBS (1 mL per 10^8^ parasites), incubated on ice for 10 min, and then centrifuged at 10,000 *g* for 30 min at 4 °C. The supernatant underwent phase partitioning by warming to 30 °C for 3 min, followed by centrifugation at 1000 *g* for 3 min at room temperature, yielding TgHyd (upper aqueous phase, ~95%) and TGMP (lower detergent phase, ~5%). TGMP was precipitated with 4 volumes of absolute ethanol (vortexed 15 s, incubated 1 h on ice), pelleted at 12,000 *g* for 20 min at 4 °C, dried (30–60 min at RT), resuspended in sterile PBS (50 µL per 3 × 10^8^ parasites), and stored at 4 °C until use. TgS was obtained by tachyzoite sonication (26 cycles of 15 s at 100 W, Branson W-185 D) in an ice bath, filtered (0.2 µm), and stored at 4 °C until use.

### 2.5. TGMP and TgS Protein Profiles

TGMP and TgS were analysed by SDS-PAGE using an 8–18% gradient running gel. The protein molecular weight (MW) standards used were from Bio-Rad (Precision Plus, Hercules, CA, USA) or Santa Cruz Biotechnology (broad-range marker). Samples containing 8 µg of protein (quantified by the Lowry protein assay) were previously heated at 95 °C for 5 min to denature the protein and then loaded into the gel. Migration was performed in a vertical electrophoresis system (mini-PROTEAN^®^ Tetra, Bio-Rad, Hercules, CA, USA) at a constant voltage of 100 V and a fixed current of 300 mA. After sample migration, the silver nitrate staining protocol was used to visualise the protein profile as previously described [13]. The gel image was captured using the ChemiDoc Imaging System (Bio-Rad, Hercules, CA, USA), and the bands were analysed using the Image Lab software version 5.2.1 (Bio-Rad, Hercules, CA, USA). For data analysis, lanes and bands were manually selected. The molecular weight analysis and quantity tools were used as provided in the Bio-Rad instructions.

### 2.6. Bone Marrow-Derived Dendritic Cells (BMDCs)

BMDCs were harvested by flushing femurs and tibias of adult female BALB/c mice with RPMI supplemented with 1 mM sodium pyruvate, 2 mM L-glutamine, 10% (*v*/*v*) inactivated FBS, 100 U ml^−1^ penicillin, and 100 µg ml^−1^ streptomycin (complete RPMI). After flushing, cells were cultured in 6-well plates at a density of 10^6^ cells/mL using complete RPMI, supplemented with HEPES buffer (20 mM), β-mercaptoethanol (50 µM), and GM-CSF (20 ng/mL; supplemented RPMI; day 0 of differentiation). Cells were incubated for 3 days at 37 °C, 5% CO_2_. On day 3, the medium was replaced with an equal volume of fresh, supplemented RPMI. On days 6 and 8, half the supplemented RPMI was replaced with fresh supplemented RPMI containing 20 ng/mL GM-CSF. On day 10, BMDC were recovered from the supernatant, followed by extended washing with warm RPMI to recover semi-adherent BMDC. Supernatants were centrifuged at 300 *g* for 10 min. Cells were washed, and viable cells were counted using the trypan blue exclusion method. BMDC were used for the viability assay, for analysis of activation marker expression upon in vitro stimulation with TGMP or TgS, and for a co-culture assay with T cells from immunised animals to determine the proliferative capacity of primed T cells, as described below.

### 2.7. Cell Viability Assay

Cell viability was assessed by the thiazolyl blue tetrazolium bromide (MTT) reduction assay using spleen cells obtained from naïve BALB/c mice, as previously described [14], and BMDC obtained as described above. Briefly, cells were seeded in 96-well plates at a density of 1 × 10^5^ cells/well. TGMP or TgS were added at 0.5–50 × 10^3^ ng/mL and resuspended in supplemented RPMI. After 24 h of treatment, 20 µL of MTT solution (Thermo-Fisher, Waltham, MA, USA) was added to each well, and the plates were incubated at 37 °C and 5% CO_2_ for 3 h. Formazan crystals were solubilised by a DMSO and isopropanol mixture (3:1). Absorbance was measured at 540 nm using a Synergy™ HTX multi-mode microplate reader (BioTek Instruments, Winooski, VT, USA). Cell viability percentage was calculated by comparing the mean absorbance values of triplicate samples from treated and untreated control cells.

### 2.8. BMDC Activation Profile by Flow Cytometry

BMDC were seeded at 2 × 10^5^ cells/well in a round 96-well plate. Cells were stimulated with TGMP (4 µg/mL), TgS (4 µg/mL), and LPS (positive control; 0.1 µg/mL; Sigma-Aldrich, St. Louis, MO, USA) with or without Polymixin B (8 µg/mL; Sigma-Aldrich, St. Louis, MO, USA) for 4 and 16 h at 37 °C in 5% CO_2_. The supernatant was discarded after centrifugation at 630 *g* for 2 min at 8 °C. Cells were incubated with APC eFluor 780 fixable viability dye (FVD) for 15 min at 4 °C. After washing with PBS, monoclonal antibodies (mAbs) specific for surface markers were added to the cells and incubated on ice for 25 min, protected from light. The following mAbs were used: Alexa Fluor 647 anti-mouse Ly6G Ab (clone 1A8); eFluor 450 anti-mouse CD11c (clone N418); PerCP/Cy5.5 anti-mouse F4/80 (clone BM8); PE anti-mouse I-A/I-E (MHC II) Ab (clone M5/114.15.2); FITC anti-mouse CD80 (clone B7-1); PE/Cy7 anti-mouse CD86 (clone GL1). Cells were fixed in PBS containing 2% paraformaldehyde (PBS-2% PFA) and washed with FACS buffer (2% foetal bovine serum (FBS) in 10 mM sodium azide in PBS). Each cell suspension was filtered into 5 mL round-bottom polystyrene test tubes using a cell strainer (Corning Falcon, Corning, NY, USA). Cell acquisition was performed in a BD FACSCanto™ II cytometer (BD Biosciences, Franklin Lakes, NJ, USA) using FACS Diva™ software version 9.0.2. Fluorescence minus one (FMO) and single-staining experiments were performed to ensure accurate interpretation and analysis of data. Data were analysed using FlowJo version 10.8.1 software (Tree Star Inc., Ashland, OR, USA). The gating strategy used is shown in Figure S1. Cells were gated based on forward scatter/side scatter (FSC-A/SSC-A), and the singlets were gated according to FSC-A vs FSC-H. Viable dendritic cells (DCs) were defined as CD11c^+^F4/80^−^Ly6G^−^FVD^−^. The number of events acquired per sample was 50,000 for the CD11c^+^F4/80^−^Ly6G^−^FVD^−^ population. The expression of the activation markers was evaluated by the mean fluorescence intensity (MFI) for MHC II, CD80, or CD86 in the CD11c^+^Ly6G^−^F4/80^−^FVD^−^ population.

### 2.9. Mouse Immunisation, Infection, and Sample Collection

Mice were assigned to groups and i.n. immunised twice at three-week intervals with: CpG (ODN 1826 VacciGrade, Invivogen, San Diego, CA, USA) at 0.25 mg/mL (5 μg per animal; 0.2 mg/Kg) or 0.5 mg/mL (10 μg per animal; 0.4 mg/Kg) plus TGMP at 0.5 mg/mL (10 µg per animal; 0.4 mg/Kg) or 1.5 mg/mL (30 µg per animal; 1.2 mg/Kg), all resuspended in saline solution (0.9% NaCl; TGMP+CpG group), or CpG alone at 0.25 mg/mL or 0.5 mg/mL resuspended in saline solution (CpG group, sham-immunised animals). The total number of animals used for each group and experiment is detailed in Table S1. The animals were i.n. immunised with 20 µL of the formulation distributed through both nostrils, on day 0, under light isoflurane anaesthesia. This procedure was repeated three weeks after the first immunisation (corresponding to the booster immunisation). Two weeks after the booster immunisation, sera were collected from mice from experiments #1, #3, #4, and #5.

The blood was collected from a tail puncture after light isoflurane anaesthesia. Blood (approximately 100 µL) was collected and left at room temperature for 1 h to clot. The samples were centrifuged at 2700 g for 20 min at 4 °C, and serum was collected into labelled tubes and stored at −80 °C for later quantification of TGMP-specific IgG1 and IgG2a antibodies and Western blotting.

In in vivo infection experiments, 3, 9, and 13 weeks after booster immunisation, animals were intraperitoneally (i.p.) infected with 5 × 10^3^ viable tachyzoites (inoculum volume of 200 μL). For inoculum preparation, parasites were isolated as previously described and resuspended in saline to a final concentration of 2.5 × 10^4^ parasites/mL. Five days post-infection (dpi) or one day dpi, mice were sacrificed. Peritoneal exudate cells (PECs) were collected by washing the peritoneal cavity with 3 mL of PBS/10% SBF. These cells were then centrifuged at 4000 *g* for 10 min, and the cell pellet was stored at −80 °C for later quantification of parasite load. Spleen, liver, lungs, heart, kidney, and brain were collected and stored at −80 °C for later quantification of parasite load.

Spleens were collected, processed as previously described [14]. Briefly, cell suspensions were prepared by gentle mechanical dissociation in PBS with 3% FCS, erythrocytes were removed with an ammonium chloride-based lysis buffer, and the remaining cells were resuspended in supplemented DMEM and counted for viability by trypan blue exclusion using a Neubauer chamber and analysed for T cells by flow cytometry in immunisation and infection experiments.

Intestinal lavage fluids (ILFs) were collected from the intestine after successive lavages with PBS containing cOmplete^™^ Mini, EDTA-free Protease Inhibitor Cocktail (Roche, Basel, Switzerland). Briefly, PBS containing protease inhibitors was passed several times through the intestinal lumen to remove all mucus and faecal content and then centrifuged at 4500 *g* for 15 min at 4°C. Supernatant was collected after centrifugation at 10,000 *g* for 1 h at 4°C and stored at −80°C until quantification of TGMP-specific IgA antibody. Blood samples were collected before infection (mice were placed in an induction chamber filled with isoflurane vapour until they reached a surgical plane of anaesthesia, as indicated by loss of righting reflex and slowed breathing; the tail vein was accessed for blood collection using a needle), and after infection at the time of sacrifice. Sera were obtained as previously described and stored at −80 °C for later detection of TGMP-specific IgG1 and IgG2a antibodies.

### 2.10. Immunoproteomic Analysis of TGMP

TGMP immune recognition was analysed by Western blot. After an SDS-PAGE using an 8–18% gradient gel, as previously described, proteins were transferred to a nitrocellulose membrane (0.45 Micron; 47 mm; Advantec, Chiyoda, Tokyo, Japan) using the Trans-Blot Turbo transfer system (Bio-Rad, Hercules, CA, USA). After blocking with 5% non-fat milk diluted in T-TBS solution (10 mM Tris Base; 50 mM NaCl; 0.1% Tween 20) (M-TTBS), the membrane was incubated overnight at 4 °C with the pooled sera from nine CpG- (10 μg CpG/animal) or TGMP+CpG (10 μg CpG + 30 μg TGMP/animal)-immunised animals at a dilution of 1:5000, using 5% M-TTBS. After being washed with T-TBS, the membrane was incubated with horseradish peroxidase-labelled goat anti-mouse IgG antibody (Southern Biotech, Birmingham, AL, USA) at a 1:2000 dilution for one hour at room temperature. The immunoreactive proteins were detected using ECL Western blotting detection reagent and analysed on the ChemiDoc Touch Imaging System (Bio-Rad, Hercules, CA, USA). Molecular weights of the detected bands were determined using Santa Cruz Biotechnology’s (Dallas, TX, USA) Broad Range Markers (sc-2361).

To perform immunoprecipitation of immunodominant TGMP, an 8–18% gradient SDS-PAGE gel was performed as previously described. After protein staining with Coomassie blue, the protein bands corresponding to the molecular weights previously identified by Western blot, were excised, and proteins were extracted from the polyacrylamide gel by the addition of elution buffer (50 mM Tris-HCl, 150 mM NaCl, 0.1 mM EDTA, pH 7.5) and incubated at 30 °C overnight, with gentle agitation. Subsequently, the proteins were precipitated with absolute ethanol and, after drying, resuspended in binding buffer (BB; 0.01 M sodium phosphate, pH 7.0, and 0.15 M sodium chloride). Agarose beads coated with Protein G (Invitrogen, Carlsbad, CA, USA) were equilibrated with BB, then incubated with sera from TGMP+CpG-immunised mice for 1 h at room temperature. After washing with BB, antibody-coated agarose beads were incubated with the protein solution extracted from the polyacrylamide gel for 1 h at room temperature with gentle continuous agitation. After incubation, the column was washed with BB and incubated with the elution buffer (0.1 M glycine HCl, pH 2.6) while shaking for 2 min. Following centrifugation, the eluted proteins were collected, resuspended in 1 M Tris base, and stored at −80 °C until further analysis. Protein identification and quantification were carried out by nanoscale liquid chromatography-tandem mass spectrometry (nanoLC-MS/MS), as previously described [15]. Data acquisition was managed using Tune 2.11 software (Thermo Scientific, Bremen, Germany), and protein identification was performed using Proteome Discoverer (v2.5, Thermo Scientific) against the UniProt 2021_03 database for *T. gondii* (ATCC 50611/strain Me49).

### 2.11. Antibody Quantification by Enzyme-Linked Immunosorbent Assay (ELISA)

The quantification of TGMP-specific serum IgG1 and IgG2 and intestinal lavage fluid IgA was performed by ELISA. Ninety-six-well flat-bottom microtiter plates (Nunc MaxiSorp plates, Thermo Fisher Scientific, Waltham, MA, USA) were coated overnight at 4 °C with TGMP (5 µg/mL) in PBS. The plate was washed with TST buffer (10 mM Tris, pH 8.0; 150 mM NaCl; 0.005% Tween-20) and then blocked in TST buffer containing 2% BSA for one hour at room temperature. The solution was discarded, and 50 μL of the sample, diluted in TST with 1% BSA (TST/1% BSA), was added and incubated for 1 h at room temperature. Six serial 1:3 dilutions were made, starting at 1:90 for serum and 1:10 for ILF. After incubation, the plate was washed 3 times with TST buffer, and the secondary antibody was added and incubated for one hour at room temperature. For IgG1 and IgG2a, alkaline phosphatase (AP)-goat anti-mouse IgG1 (Southern Biotech, Birmingham, AL, USA) and AP-goat anti-mouse IgG2a (Southern Biotech, Birmingham, AL, USA), respectively, were used at a fold dilution of 1:1000 in TST/1% BSA. For IgA, AP-goat anti-mouse IgA (Southern Biotech, Birmingham, AL, USA) was used at a dilution of 1:500 in TST/1% BSA. The plates were then washed three times with TST. The substrate was prepared by dissolving one p-nitrophenyl phosphate substrate tablet (Sigma-Aldrich, St. Louis, MO, USA) in 5 mL of AP buffer (50 mM Na_2_CO_3_, 1 mM MgCl_2_). Then, 50 μL was added per well and incubated in the dark at room temperature for 20 min. The reaction was stopped with 50 μL per well of 0.1 M EDTA, pH 8.0, and the colourimetric signal was quantified by measuring absorbance at 405 and 570 nm using the Synergy HTX Multi-Mode Microplate Reader and Gen5 2.0 Data Analysis Software (both from BioTek Instruments, Winooski, VT, USA).

### 2.12. Antigen-Specific T Cell Proliferation by Flow Cytometry

A co-culture assay was performed using BMDC generated as described above and spleen T cells from CpG- or TGMP+CpG-immunised animals, prepared as previously described [14]. BMDCs were seeded in 96-well round-bottom plates at a density of 2.5 × 10^4^ cells/well, and stimulated with TGMP (4 µg/mL) or TgS (4 µg/mL). A positive control was included, consisting of overnight stimulation with purified, no-azide/low-endotoxin anti-CD3e (1 µg/mL; clone 145-2C11; BD Biosciences, Franklin Lakes, NJ, USA). Splenic T cells were isolated from TGMP+CpG- or CpG-immunised mice (15 weeks after boost immunisation) using the MojoSort™ Mouse CD3 T Cell Isolation Kit (BioLegend, San Diego, CA, USA), according to the manufacturer’s instructions, yielding untouched CD3^+^ T cells. Isolated T cells were labelled with CellTrace™ Violet (CTV) stain (Thermo Fisher Scientific, Waltham, MA, USA) at 1:500 for 20 min at 37 °C in the dark. To neutralise the free reactive dye, T cells were resuspended in complete RPMI. BMDCs (2.5 × 10^4^ cells/well) were co-cultured with CTV-labelled T cells (1 × 10^5^ cells/well) in 96-well round-bottom plates at a 1:4 ratio (BMDC: T cells) in complete RPMI for 5 days at 37 °C, 5% CO_2_. Control wells included T cells cultured with unstimulated BMDC. After incubation, the supernatants were stored for future analyses upon centrifugation at 630 *g* for 2 min at 8 °C. Cells were incubated with APC eFluor 780 fixable viability dye (FVD; Invitrogen, Invitrogen, Carlsbad, CA, USA) for 15 min at 4 °C. After washing with PBS, antibodies specific for T cell surface markers were added to the cells, which were then incubated for 25 min on ice, protected from light. The following mAbs were used: APC anti-mouse CD3ε (clone 145-2C11; BioLegend, San Diego, CA, USA), PE anti-mouse CD4 (clone RM4-5; BioLegend, San Diego, CA, USA), FITC anti-mouse CD8 (clone 53-6.7; BioLegend, San Diego, CA, USA). After incubation, the cell suspension was washed with FACS buffer and fixed in PBS 2% paraformaldehyde (PFA) for 20–25 min at room temperature. After being washed and resuspended in FACS buffer, cells were filtered using a 5 mL round-bottom polystyrene test tube with a cell strainer (Corning). Cell acquisition was performed using BD FACSymphony A1 (BD Biosciences, NJ, USA). Fluorescence minus one (FMO) and single-staining experiments were performed to ensure accurate interpretation and analysis of data. Data were analysed using FlowJo version 10.8.1 software (Tree Star Inc., Ashland, OR, USA). The gating strategy is indicated in the Figure S2. Cells were gated based on forward scatter/side scatter (FSC-A/SSC-A), and the singlets were gated according to FSC-A vs FSC-H. The number of events acquired for each sample was 30,000 in the singlets and FVD^−^ cell population. The CD4^+^ T cells were defined as CD3^+^CD4^+^CD8^−^, and CD8^+^ T cells were defined as CD3^+^CD8^+^CD4^−^. The percentage of proliferating CD4^+^ and CD8^+^ T cells was determined by the reduction in CTV fluorescence intensity, with each cell division corresponding to a halving of fluorescence.

### 2.13. DNA Extraction and Quantitative Real-Time PCR (Q-PCR)

Genomic DNA (gDNA) was extracted from spleen, kidney, liver, lung, heart, brain, and PEC by the phenol-chloroform method, followed by precipitation with ammonium acetate/ethanol. Tissues were homogenised with 2 mL of lysis buffer (75 mM NaCl and 25 mM EDTA), and the PEC pellet was homogenised in 500 μL of lysis buffer. From each tissue, 500 μL aliquots were collected, to which 50 μL of 10% SDS (final concentration 1% SDS) and 10 μL of 25 mg/mL Proteinase K (final concentration 0.5 mg/mL) were added and incubated overnight at 55 °C. Then, 500 μL of phenol-chloroform (Merck) was added to the samples, which were centrifuged at 6000 *g* for 15 min at 4 °C. The aqueous phase was transferred to another microtube, to which 500 μL of phenol-chloroform was added, and the samples were then centrifuged for 15 min at 6000 *g* at 4 °C. The resulting aqueous phase was transferred to another microtube, to which 100% ethanol and 7.5 M ammonium acetate were added in the following proportions: ethanol, 2× the volume of the aqueous phase; ammonium acetate, 1/3 the volume of the aqueous phase. The mixture was incubated for 1 h at −80 °C, then centrifuged at 16,000 *g* for 15 min at 4 °C. The pellet (gDNA) was washed with 500 μL of 70% ethanol and centrifuged at 16,000 *g* for 10 min at 4 °C. The supernatant was discarded, and the samples were incubated at 37°C for 30 min to allow the remaining ethanol to evaporate. gDNA was resuspended in 200 μL of ultra-pure water and stored overnight at 4 °C to facilitate solubilization. Samples were quantified using the Thermo Scientific Nanodrop 1000 spectrophotometer. The DNA concentration was adjusted to 50 ng/mL for PE and 200 μg/mL for the other tissues as a template DNA.

*T. gondii* genomic DNA was detected by qPCR using primers and a probe targeting the SAG1 gene (GenBank: X14080), generating an amplicon of approximately 100 bp. Reactions were performed with 1 μL of DNA in a total volume of 10 μL containing 0.2 μmol/L of each primer (SAG-1 forward: CCAGAGCCTCATCGGTCGTC; SAG-1 reverse: GGGTCCTTCCGCAG ACAAC), 0.2 μmol/L of probe (6FAM-CTGTyTGCACCGTAGGAGCACCT-BBQ; Tib Molbiol), and Kapa probe Fast qPCR Master Mix (Kapa Biosystems). The lowercase “y” in the probe sequence represents an IUPAC degenerate base code for a pyrimidine, indicating an equal mixture of Cytosine (C) and Thymine (T) at that specific position. The qPCR cycling conditions consisted of an initial denaturation at 95°C for 3 min, followed by 40 cycles of 95 °C for 3 s and 60 °C for 30 s for annealing and extension. Quantification was performed using a standard curve generated from *T. gondii* tachyzoite gDNA, ranging from 5 × 10^2^ to 5 × 10^−2^ ng (corresponding to 2 × 10^7^ to 2 × 10^3^ parasites) in each run. Fluorescence data were acquired using the StepOne Plus system and analysed with StepOne Software v2.3 (Applied Biosystems by Life Technologies, Bleiswijk, The Netherlands).

### 2.14. Interferon-Gamma (IFN-γ) Quantification by ELISA

Splenocytes from immunised mice (CpG or TGMP+CpG) were processed as described [14]. Spleen cells were plated at 2 × 10^5^ cells per well in 96-well round-bottom plates and stimulated in triplicate with one of the following conditions: 4 μg/mL TGMP, 4 μg/mL TgS, or 1 μg/mL Concanavalin A (ConA; positive control). Cells were incubated at 37 °C in 5% CO_2_ for 48 h. Supernatants were collected and stored at −80 °C until cytokine analysis. According to the manufacturer’s instructions, IFN-γ concentrations in culture supernatants were measured using the Mouse IFN-γ (homodimer) Uncoated ELISA Kit (Thermo Fisher Scientific, Waltham, MA, USA). Briefly, 96-well plates were coated overnight at 4 °C with the provided capture antibody. After washing and blocking, 100 μL of standards, controls, or supernatant samples were added in triplicate and incubated for 2 h at room temperature. Plates were washed, incubated with the biotinylated detection antibody for one hour, then incubated with streptavidin-HRP for 30 min. After a final wash, 3,3′,5,5′-Tetramethylbenzidine substrate was added, and the reaction was stopped with the stop solution (1M H_3_PO_4_). Absorbance was measured at 450 nm using the Synergy HTX Multi-Mode Microplate Reader and using the Gen5 2.0 Data Analysis Software (both from BioTek Instruments, Winooski, VT, USA). IFN-γ concentrations were determined by interpolation from a standard curve generated with recombinant mouse IFN-γ standards.

### 2.15. Evaluation of the Memory Phenotype of Spleen TCD4^+^ and TCD8^+^ Cells

As previously described, a single-cell suspension was prepared from the spleen of each mouse [14]. A total of 1 × 10^6^ spleen cells were added to each well of a round 96-well plate. The supernatant was discarded after centrifugation at 630 *g* for 2 min at 8 °C. Cells were incubated with APC eFluor 780 fixable viability dye (FVD) for 15 min at 4 °C. After washing with PBS, antibodies specific for surface markers were added to the cells and incubated for 25 min on ice, protected from light. The following mAbs were used: APC anti-mouse CD3 (clone 17A2), BV421 anti-mouse CD4 (clone RM4-5), PerCP/Cy 5.5 anti-mouse CD8 (clone 53-6.7), PECy7 anti-mouse CD44 (clone IM7), and PE anti-mouse CD62L (clone MEL14) (all from eBioscience, San Diego, CA, USA). After incubation, the cell suspension was washed with FACS buffer, fixed with PBS-2% PFA for 20–25 min at room temperature. After being washed twice and resuspended in FACS buffer, cells were filtered using 5 mL round-bottom polystyrene test tube with a cell strainer (Corning Falcon). Cell acquisition was performed using BD FACSCanto™ II cytometer (BD Biosciences, San Diego, NJ, USA). Fluorescence minus one (FMO) and single-staining experiments were performed to ensure accurate interpretation and analysis of data. Data were analysed using FlowJo version 10.8.1 software (Tree Star Inc., Ashland, OR, USA). The gating strategy is indicated in the Figure S3. Cells were gated based on forward scatter/side scatter (FSC-A/SSC-A), and the singlets were gated according to FSC-A vs FSC-H. The number of events acquired for each sample was 50,000 in the singlet FVD-cell population. CD4^+^ T cells were defined as CD3^+^CD4^+^CD8^−^, and CD8^+^ T cells were defined as CD3^+^CD8^+^CD4. The CD4^+^ T or CD8^+^ T cells displaying a CD44^+^CD62L^+^ phenotype were considered central memory TCD4^+^ or TCD8^+^ cells, and those displaying a CD44^+^CD62L^–^ phenotype were considered effector memory TCD4^+^ or TCD8^+^ cells.

### 2.16. Evaluation of Intracellular IFN-γ, IL-17, and Granzyme B (GranzB) by T Cells

Spleen cells (1 × 10^6^) were stimulated with TGMP (4 µg/mL) and TgS (4 µg/mL) for 16 h at 37 °C, 5% CO_2_. The positive control consisted of the cell stimulation with Phorbol 12-myristate 13-acetate (50 ng/mL) plus Ionomycin (500 ng/mL) (PMA/IONO; both from Sigma-Aldrich) for 4 h at 37 °C, 5% CO_2_. Brefeldin A (100 μg/mL) was added to each well and incubated for 2 h at 37 °C and 5% CO_2_. The supernatant was discarded after centrifugation at 630 *g* for 2 min at 8 °C. Cells were incubated with APC eFluor 780 fixable viability dye (FVD) for 15 min at 4 °C. After washing with PBS, antibodies specific for surface markers were added to the cells and incubated for 25 min on ice, protected from light. The following mAbs were used for surface antigen staining: eFluor 506 anti-mouse CD3 (clone 17A2), BV421 anti-mouse CD4 (clone RM4-5), PerCP/Cy5.5 anti-mouse CD8 (clone 53-6.7), PECy7 anti-mouse CD44 (clone IM7) (all from eBioscience, CA, USA). After incubation, the cell suspension was washed with FACS buffer, fixed with PBS-2% PFA for 20–25 min at room temperature. After being washed twice with FACS buffer, cells were permeabilised with FACS buffer plus 0.5% Saponin (Sigma) for 10 min at room temperature. To avoid nonspecific Fcγ receptor binding, cells were preincubated with anti-mouse CD16/CD32 (clone S17011E; BioLegend, San Diego, CA, USA) for 10–15 min at 4 °C. The following mAbs were used for intracellular labelling: PECy7 anti-mouse IFN-γ (XMG1.2); PE anti-mouse IL-17A (TC11-18H10.1); and eFluor 450 anti-mouse GranzB (NGZB) (all from BioLegend, San Diego, CA, USA). Cells were incubated for 30 min in the dark at room temperature. After being washed and resuspended in FACS buffer, cells were filtered using a 5 mL round-bottom polystyrene test tube with a cell strainer (Corning Falcon). Cell acquisition was performed using a BD FACSCanto™ II cytometer (BD Biosciences). Fluorescence minus one (FMO) and single-staining experiments were performed for accurate data interpretation and analysis. Data were analysed using FlowJo version 10.8.1 software (Tree Star Inc., Ashland, OR, USA). Cells were gated based on forward scatter/side scatter (FSC-A/SSC-A), and the singlets were gated according to FSC-A vs FSC-H. The number of events acquired for each sample was 50,000 in the singlets FVD^−^ cell population. The gating strategy is indicated in the Figure S4. The CD4^+^ T cells were defined as CD3^+^CD4^+^CD8^−^, and CD8^+^ T cells were defined as CD3^+^CD8^+^CD4^−^. Activated CD4^+^T or CD8^+^T cells were evaluated based on CD44 expression (CD3^+^CD4^+^CD44^+^ or CD3^+^CD8^+^CD44^+^). The IFN-γ, IL-17 and GranzB were evaluated in the CD4^+^T (CD3^+^CD4^+^IFN-γ^+^, CD3^+^CD4^+^IL-17^+^, and CD3^+^CD4^+^GranzB^+^) or CD8^+^T cells (CD3^+^CD8^+^IFN-γ^+^, CD3^+^CD8^+^IL-17^+^ and CD3^+^CD8^+^GranzB^+^) and also in the activated CD4^+^T (CD3^+^CD4^+^ CD44^+^IFN-γ^+^, CD3^+^CD4^+^ CD44^+^IL-17^+^, and CD3^+^CD4^+^ CD44^+^GranzB^+^) or activated CD8^+^ T cells (CD3^+^CD8^+^CD44^+^IFN-γ^+^, CD3^+^CD8^+^CD44^+^IL-17^+^ and CD3^+^CD8^+^CD44^+^GranzB^+^).

### 2.17. Statistical Analyses

**Figure 1:**
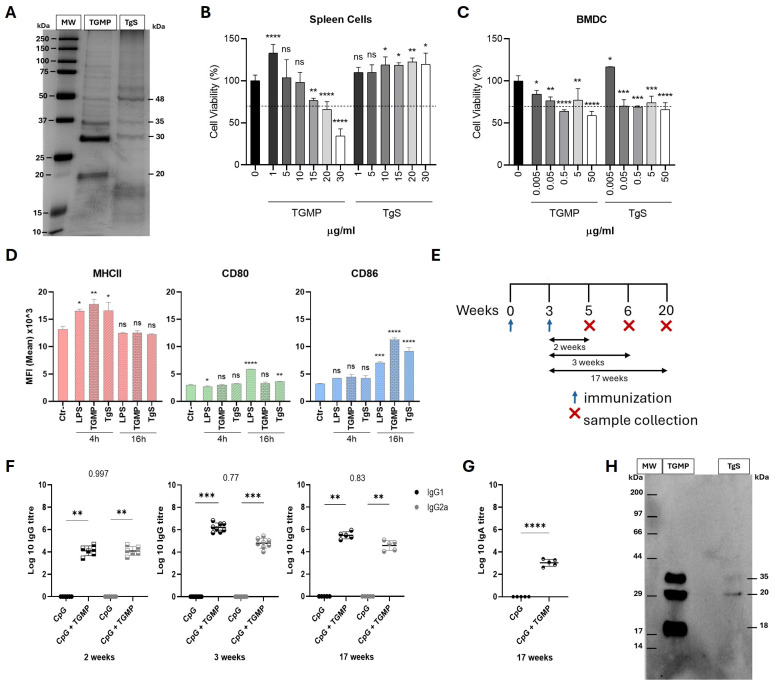

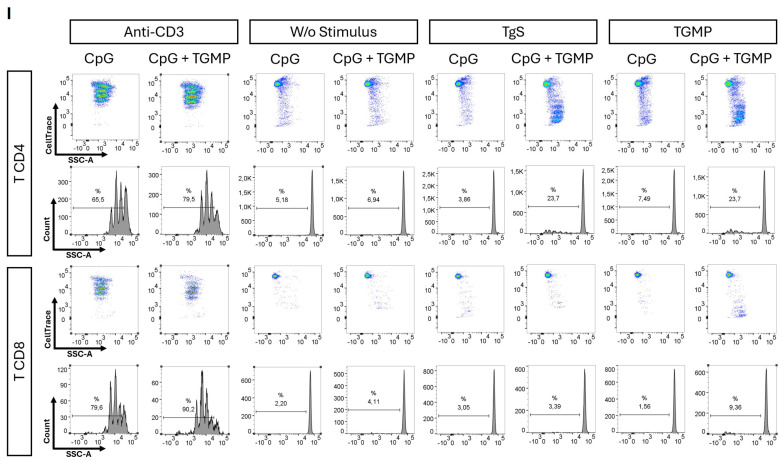
*T. gondii* membrane antigen extract (TGMP) characterisation, biological evaluation, and immunomodulatory effect. (**A**) Protein profile of TGMP and whole antigen extract (TgS). Analysis by gradient SDS-PAGE. (**B**,**C**) Effect of TGMP or TgS on the cell viability percentage (%) using spleen cells and bone-marrow-derived dendritic cells (BMDC). Cells were treated with different concentrations of TGMP or TgS for 24 h. Triplicates were run for each tested concentration, and data are expressed as mean + SD (data shown is representative of three independent experiments). (**D**) Effect of TGMP and TgS on BMDC activation markers, MHCII, CD80 and CD86 after 4 and 16 h of stimulation. Lipopolysaccharide (LPS) was used as a positive control. Data are expressed as mean + SD (*n* = 3). MFI: mean fluorescence intensity. Comparisons were made between stimulated cells and unstimulated cells (Ctr-) for each time point. (**E**) Experimental design for in vivo experiments. BALB/c mice were intranasally immunised twice with CpG alone (CpG; 10 µg per animal) or CpG adjuvant plus TGMP (TGMP+CpG; 30 µg + 10 µg per animal). At 2, 3 and 17 weeks after the boost immunisation, the samples were collected for analysis. (**F**,**G**) Quantification of TGMP-specific IgG1 and IgG2a in the serum at 2, 3, and 17 weeks after the booster immunisation, and TGMP-specific IgA in the intestinal lavage fluids at 17 weeks after boost immunisation. The numbers above each graph represent the IgG2a/IgG1 ratio for each time point. Each dot represents an individual mouse, and horizontal lines correspond to the mean value in each group (2 weeks, *n* = 6; 3 weeks, *n* = 8; 17 weeks, *n* = 5). Data are expressed as mean ± SD of the log_10_ of the antibody titres. Comparisons were made between CpG and TGMP+CpG groups. (**H**) Detection of immunogenic proteins from TGMP. Western blot was performed using pooled sera from immunised BALB/c mice with TGMP+CpG, collected 3 weeks after booster immunisation. (**I**) T cell proliferation following in vitro co-culture of BMDC preloaded with TGMP or TgS and spleen T cells isolated from TGMP-immunised or sham-immunised (15 weeks after booster immunisation). Anti-CD3 stimulation was used as the positive control. Representative image from three independent experiments, each comprising a single measurement per condition. Standard protein molecular weight (MW) is expressed in kDa. * *p* < 0.05; ** *p* < 0.01; *** *p* < 0.001; **** *p* < 0.0001; ns = not significant.

**Figure 2:**
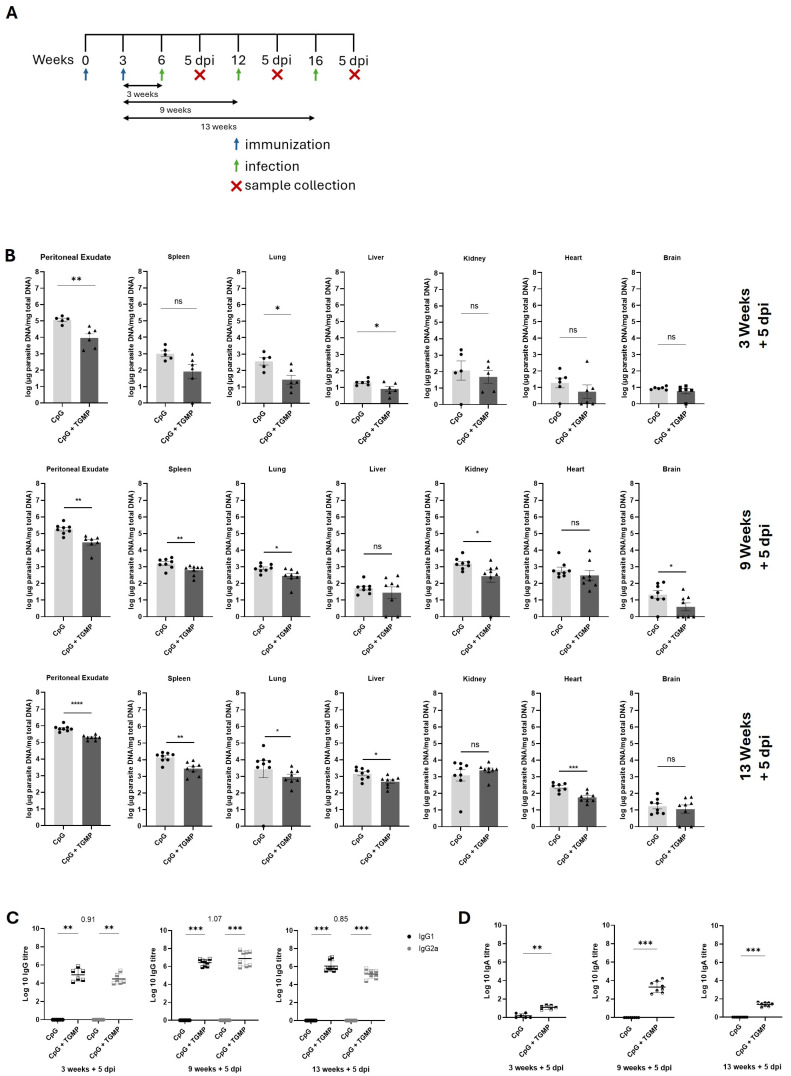
Intranasal (i.n.) immunisation with *T. gondii* membrane antigen extract (TGMP) induces partial protection against acute infection and TGMP-specific humoral responses. (**A**) Experimental design for in vivo experiments. BALB/c mice were intranasally immunised twice with CpG alone (CpG; 10 µg per animal) or CpG adjuvant plus TGMP (TGMP+CpG; 30 µg + 10 µg per animal). Mice were intraperitoneally infected with 5000 tachyzoites of the Me49 *T. gondii* strain at 3, 9, and 13 weeks after the boost immunisation. Five days post-infection (5 dpi), the samples were collected for analysis. (**B**) Protective effect of i.n. immunisation with CpG plus TGMP (TGMP+CpG) against acute *T. gondii* infection. The parasite load was quantified by quantitative PCR for *SAG-1* in peritoneal exudate cells (peritoneal exudate), spleen, lung, liver, kidney, heart, and brain. Each dot represents an individual mouse (3 weeks, n = 6; 13 and 9 weeks, n = 8). Data are expressed as mean ± SD. * *p* < 0.05; ** *p* < 0.01; *** *p* < 0.001; **** *p* < 0.0001; ns = not significant. Comparisons were made between CpG- and TGMP+CpG-immunised animals. (**C**,**D**) *T. gondii* infection does not alter the profile of humoral responses of i.n. TGMP-immunised animals. Quantification of serum TGMP-specific IgG1 and IgG2a, and TGMP-specific IgA in the intestinal lavage fluid, 5 days post-infection (5 dpi). The numbers above each graph represent the IgG2a/IgG1 ratio for each time point. Each dot represents an individual mouse, and horizontal lines correspond to the mean value in each group (3 weeks, n = 6; 9 and 13 weeks, n = 8). Data are expressed as mean ± SD of the log_10_ of the antibody titres. * *p* < 0.05; ** *p* < 0.01; *** *p* < 0.001; **** *p* < 0.0001; ns = not significant. Comparisons were made between the CpG group and the TGMP+CpG group.

**Figure 3:**
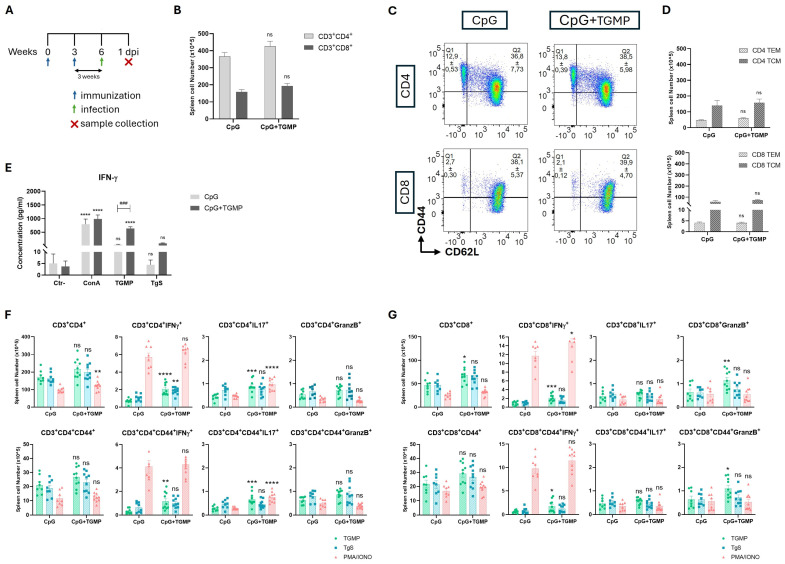
Intranasal (i.n.) immunisation with membrane antigen extract (TGMP) induces TGMP-specific T cell responses after infection. (**A**) Experimental design for in vivo experiments. BALB/c mice were intranasally immunised twice with CpG alone (CpG; 10 µg per animal) or CpG adjuvant plus TGMP (TGMP+CpG; 30 µg + 10 µg per animal). Mice were intraperitoneally infected with 5000 tachyzoites of the Me49 *T. gondii* strain at 3 weeks after the booster immunisation. One day post-infection (1 dpi), spleens were collected for analysis. (**B**) Total TCD4^+^ (CD3^+^CD4^+^) and TCD8^+^ (CD3^+^CD8^+^) spleen cell numbers. (**C**,**D**) Spleen memory phenotype of CD4^+^ and CD8^+^ T cells. (C) Representative dot plot analysis of gated CD4^+^ and CD8^+^ T cells expressing CD44 and CD62L. The numbers within dot plots correspond to mean ± SD of percentage values for the particular quadrant region in the respective CpG-immunised or TGMP+CpG-immunised groups. (**D**) Numbers of spleen CD4 T effector memory cells (CD4 TEM; CD3^+^CD4^+^CD44^+^CD62L^−^); CD4 T central memory cells (CD4 TCM; CD3^+^CD4^+^CD44^+^CD62L^+^); CD8 T effector memory cells (CD8 TEM; CD3^+^CD8^+^CD44^+^CD62L^−^) and CD8 T central memory cells (CD8 TCM; CD3^+^CD8^+^CD44^+^CD62L^+^). Data represent the mean + SEM of mice analysed individually. The results are from two experiments that yielded concordant results (n = 10 in each group). Statistical analysis was performed by 2-way ANOVA and Sidak’s multiple comparisons test. ns: not significant. Comparisons were made between spleen cells from CpG- and TGMP+CpG-immunised mice. (**E**) IFN-γ production by splenocytes from immunised and sham-immunised infected mice. IFN-γ was quantified in the supernatant of spleen cells after in vitro stimulation with TGMP (4 µg/mL) and TgS (4 µg/mL) for 72 h. Concanavalin A (Con A; 1 μg/mL) was used as a positive control. Each stimulus was tested in triplicate. Data are expressed as the mean ± SD of the values for each individual mouse. **** *p* < 0.0001; ^###^ *p* < 0.001; ns = not significant. The symbol * represents comparisons between each stimulus and the control group. (**F**,**G**) Evaluation of spleen T cell activation and responses to TGMP stimulation. Spleen cells were ex vivo stimulated with TGMP (4 µg/mL) or TgS (4 µg/mL) for 16 h. The phorbol 12-myristate 13-acetate plus Ionomycin (PMA/IONO) stimulation for 4 h was used as a positive control. (**F**) CD4^+^T cells (CD3^+^CD4^+^), CD4^+^ T cells producing IFN-γ (CD3^+^CD4^+^IFNγ^+^), IL-17 (CD3^+^CD4^+^IL17^+^) and GranzB (CD3^+^CD4^+^GranzB^+^); activated CD4^+^T cells (CD3^+^CD4^+^CD44^+^), activated CD4^+^ T cells producing IFN-γ (CD3^+^CD4^+^CD44^+^IFNγ^+^), IL-17 (CD3^+^CD4^+^CD44^+^IL17^+^) and GranzB (CD3^+^CD4^+^CD44^+^GranzB^+^). (G) CD8^+^T cells (CD3^+^CD8^+^), CD8^+^ T cells producing IFN-γ (CD3^+^CD8^+^IFNγ^+^), IL-17 (CD3^+^CD8^+^IL17^+^) and GranzB (CD3^+^CD8^+^GranzB^+^); activated CD8^+^T cells (CD3^+^CD8^+^CD44^+^), activated CD8^+^ T cells producing IFN-γ (CD3^+^CD8^+^CD44^+^IFNγ^+^), IL-17 (CD3^+^CD8^+^CD44^+^IL17^+^) and (CD3^+^CD8^+^CD44^+^GranzB^+^). Data represent the mean ^+^ SEM of mice analysed individually. The results are from two independent experiments that yielded concordant results (n = 5 in each group per experiment; n total = 10 per group). *: *p* < 0.05; **: *p* < 0.01; ***: *p* < 0.001; ****: *p* < 0.0001; ns: not significant. Comparisons were made between spleen cells from CpG- and TGMP+CpG-immunised mice.

GraphPad Prism version 9.0 (GraphPad Software, Inc., San Diego, CA, USA) was used for statistical analysis. For multiple group comparisons with one independent variable, a one-way ANOVA with a Tukey post hoc test was used (Figure 1D). For group analysis with two independent variables, two-way ANOVA with a Tukey’s post-test was performed (Figure 1B,C). Unpaired Student’s *t*-test was used to compare statistical differences between two groups, and a one-way analysis of variance (ANOVA) test and Dunn’s multiple comparisons for experiments with 3 or more groups (Figure 1F,G and Figure 2B–D). When normality or homogeneity of variances was not observed, comparisons were carried out using the Mann–Whitney test. A Shapiro–Wilk test of normality was done to decide whether to use parametric or non-parametric tests. For the data obtained by flow cytometry (Figure 3B,D,F,G) and ELISA (Figure 3E), a two-way analysis of variance (ANOVA) and Sidak’s multiple comparisons test was performed. Statistical significance was assessed for *p* ˂ 0.05.

### 2.18. Data Availability

The datasets used and/or analysed during the current study are available from the corresponding author on reasonable request.

## 3. Results

### 3.1. TGMP Extract Reveals an Enrichment of Proteins at 20, 30, and 35 kDa

The TGMP were extracted from *T. gondii* Me49 tachyzoites obtained by in vitro infection of Vero cells and isolated using size-exclusion chromatography. A gradient SDS–PAGE was used to analyse TGMP and TgS under reducing conditions. TGMP exhibited an enrichment of three protein bands with molecular weights (MW) of approximately 20, 30, and 35 kDa, compared to the TgS (Figure 1A).

### 3.2. TGMP Extract Induces the Activation of DCs

The biocompatibility of TGMP and TgS was assessed using the MTT assay with mouse spleen cells and BMDC to identify safe concentrations for the subsequent in vitro studies. Lower TGMP and TgS concentrations were tested for BMDCs than for splenocytes, as these primary cultures are more sensitive to protein antigens and more prone to cytotoxicity than the more robust splenocyte population. TGMP did not significantly affect spleen cell viability at concentrations below 10 µg/mL (Figure 1B). However, TGMP reduced BMDC viability to below 70% within the concentration range of 0.5–50 µg/mL, while remaining above 50% (Figure 1C).

The yield of viable DC (CD11c^+^F4/80^−^Ly6G^−^FVD^−^) was approximately 40%, as confirmed by flow cytometry analysis following BMDC differentiation. The activation of DC by TGMP or TgS was evaluated by measuring the surface expression of MHC II, CD80, and CD86 on viable DC upon stimulation. DCs were stimulated with TGMP or TgS for 4 h and 16 h prior to flow cytometric analysis of MHCII, CD80, and CD86 expression. These timepoints were selected based on established activation kinetics, in which MHCII surface expression increases rapidly via mobilisation of pre-existing pools and reduced internalisation, detectable within 2–6 h post-stimulation. In contrast, costimulatory molecules CD80/CD86 require prolonged signalling for maximal transcription/translation, with peak surface levels often at 12–24 h [16,17]. The results showed that TGMP and TgS significantly increased MHC II and CD86 expression on viable DCs at 4 and 16 h, respectively. These increases were comparable to those induced by the positive control (LPS). No differences were observed in CD80 expression after TGMP or TgS stimulations (Figure 1D). CD86 upregulation without CD80 is a well-documented phenomenon in *T. gondii*-stimulated DCs, as CD86 responds more robustly to parasite antigens via distinct signalling thresholds (CD86 has lower activation requirements and faster TLR/MyD88 kinetics), whereas CD80 requires stronger/prolonged stimuli, such as LPS, for equivalent induction. [18,19]. To further confirm that the increased expression of MHC II and CD86 on DC was not due to endotoxin contamination, polymyxin treatment was conducted simultaneously with the various stimuli. The reversion of the stimulatory effect was only observed for LPS stimulation (Figure S5).

### 3.3. Prime-Boost Immunisation Induces TGMP-Specific Antibodies

The immunisation strategy (Figure 1E) was based on a method previously used in the murine model to vaccinate against *N. caninum*, a parasite closely related to *T. gondii* [11]. Serum TGMP-specific IgG1 and IgG2a levels were quantified at short (2 and 3 weeks) and long term (17 weeks) after the booster immunisation (Figure 1F). Intestinal TGMP-specific IgA levels were quantified at long term (Figure 1G). The obtained results indicated a significant increase in TGMP-specific IgG1 and IgG2a in animals immunised with TGMP+CpG, compared with the sham-immunised mice receiving CpG alone (Figure 1F). Independent experiments in which varying amounts of TGMP and CpG were used showed that all combinations tested TGMP+CpG (10 µg + 5 µg, 30 µg + 5 µg, 10 µg + 10 µg) induced a significant increase in TGMP-specific circulating IgG1 and IgG2a compared to the sham-immunised animals (Figure S6). In fact, a mixed IgG1 and IgG2a response was observed at all the time points tested, along with the varying amounts of TGMP and CpG used in i.n. immunisation (Figure 1F and Figure S6B). However, it was noted that when using 5 µg of CpG in the immunisation, the IgG2a/IgG1 ratios shifted to values above 1.0, indicating that the IgG2a response was superior than that of IgG1 (Figure S6B). A significant increase in TGMP-specific IgA in the ILF from TGMP+CpG-treated animals was observed compared to sham-immunised animals (Figure 1G), as well as when 5 µg of CpG was used (Figure S6C).

### 3.4. Nine Major Immunogenic Proteins Identified in the TGMP Extract

To identify the principal immunogenic proteins composing TGMP, a Western blot analysis was performed using sera from TGMP+CpG or sham-immunised mice. Three prominent bands were detected with molecular weights of approximately 20, 30, and 35 kDa, when using sera from mice immunised with TGMP+CpG (Figure 1H), and these bands were consistently detected across biological replicates. The sera from the sham-immunised mice did not significantly recognise TGMP proteins (Figure S6D). The raw data from the Western blot experiment are provided in Figure S7. The protein molecular weights of the major bands detected by Western blot and stained SDS-PAGE (Figure 1A,H) were similar, probably corresponding to the same proteins. The bands were excised, proteins were extracted from the SDS-PAGE gel, pooled, and immunoprecipitated using Protein G-coated beads pre-incubated with sera from TGMP+CpG-immunised mice. The eluted proteins were then analysed by mass spectrometry. The results indicated that the eluted fraction consisted of proteins from mice (*Mus musculus*) and the *T. gondii* Me49 strain (File S1). Table 1 lists nine *T. gondii* Me49 proteins identified by mass spectrometry that presented sequence coverage ≥10%, unique peptide ≥ 2, and score sequest HT ≥ 1.5. The higher the score HT, the higher the certainty in the protein identification. The following proteins are listed from the highest to the lowest sequence coverage: dense granule protein 7 (GRA7; https://www.uniprot.org/uniprotkb/O00933/entry; accessed on 19 January 2022), SAG-related sequence 25 (SRS 25; https://www.uniprot.org/uniprotkb/S8FBZ9/entry; accessed on 19 January 2022), dense granule protein 2 (GRA2, https://www.uniprot.org/uniprotkb/P13404/entry; accessed on 19 January 2022), SAG-related sequence SRS34A (SRS34A; https://www.uniprot.org/uniprotkb/A0A125YIJ3/entry; accessed on 19 January 2022), Toxofilin (https://www.uniprot.org/uniprotkb/S8G4J8/entry; accessed on 19 January 2022), SAG-related sequence 29B (SRS29B, https://www.uniprot.org/uniprotkb/A0A125YP09/entry; accessed on 19 January 2022), nascent polypeptide-associated complex (NAC) domain-containing protein (https://www.uniprot.org/uniprotkb/A0A125YY47/entry; accessed on 19 January 2022), NAC subunit beta (https://www.uniprot.org/uniprotkb/S8GBN3/entry; accessed on 19 January 2022), and microneme protein 10 (MIC10; https://www.uniprot.org/uniprotkb/A0A125YLT9/entry; accessed on 19 January 2022).

GRA2 and GRA7 have been recognised as having a significant role in both the acute and chronic phases of *T. gondii* infection, making them potential candidates for an effective vaccine [20,21]. NAC-related proteins were detected, suggesting a possible parasite-associated homolog, given that NAC is typically a host-associated complex, although additional validation is needed to confirm their role and localisation. The Uniprot data available for SRS25, SRS29B, SRS34A, Toxofilin, NAC domain-containing protein, NAC subunit beta, and MIC 10 consisted of unreviewed entries.

### 3.5. TGMP-Loaded BMDC Induces T Cell Proliferation

To assess the proliferative capacity of TGMP-primed T cells in response to TGMP antigenic presentation by DCs, co-culture assays were conducted using BMDC preloaded with TGMP or TgS and spleen T cells isolated from TGMP-immunised or sham-immunised animals. A positive control, consisting of T cells stimulated with anti-CD3 mAb, was included. As anticipated, a substantial increase in both CD4^+^ and CD8^+^ T cells in response to anti-CD3 stimulation was observed in both TGMP- and sham-immunised mice, indicating that the T cells were functional and responsive (Figure 1I). TGMP- or TgS-preloaded DCs induced detectable CD4^+^ T cell proliferation using T cells obtained from the spleens of mice immunised with TGMP+CpG as responders, but not from those immunised with CpG alone (Figure 1I). These results indicate that prior immunisation with TGMP induced TGMP-specific and TgS-specific CD4^+^ memory T cells. A lower level of CD8^+^ T cell proliferation was observed using the same experimental conditions (Figure 1I). These results altogether indicate that the antigen recall response was predominantly restricted to the CD4^+^ T cell compartment.

### 3.6. Mice i.n.-Immunised with TGMP+CpG Presented a Lower Parasitic Burden Upon Infection with T. gondii

The parasite load in the peritoneal exudate (PE) cells and organs (spleen, lung, liver, kidney, heart, and brain) collected from acutely infected animals (5 dpi) was assessed 3, 9, and 13 weeks after the boost immunisation (Figure 2A,B). The parasite load was assessed at 5 days post-infection, as the mice had reached the humane endpoint by the subsequent day, precluding further monitoring (Table S2). As demonstrated, immunisation with TGMP+CpG (30 µg + 10 µg) significantly reduced parasite load in the PE and lungs at 3, 9, and 13 weeks after the boost compared with sham-immunised animals. In the spleen, a significant decrease in parasitic burden was observed 9 and 13 weeks after the boost immunisation. A tendency toward reduced parasitic load was observed in the liver, kidney, heart, and brain, although it did not reach statistical significance. The parasite load was also determined in the PE and tissues of mice immunised with different combinations of TGMP+CpG (10 µg + 5 µg; 30 µg+ 5 µg; 10 µg + 10 µg, Figure S8A). In accordance with the results presented above, the parasite load was significantly reduced in the TGMP+CpG group compared to sham-immunised animals for all the TGMP+CpG combinations used. A decrease in the spleen, lung, and liver was also found, although it was not consistently significant (Figure S8B). Taken together, these results showed a protective effect against *T. gondii* induced by i.n. immunisation with TGMP+CpG in both the short and long term. 

### 3.7. Raised TGMP-Specific Antibody Levels Induced by Immunisation Are Sustained Following Infection

The levels of TGMP-specific IgG1 and IgG2a were measured in infected mice, 3, 9 and 13 weeks after booster immunisation with TGMP+CpG (30 µg TGMP + 10 µg CpG) or CpG alone (10 µg) (Figure 2C). The levels of IgG1 and IgG2a were similar to those previously quantified before infection, and a significant increase was observed in TGMP-specific IgG1 and IgG2a titres in the TGMP+CpG-immunised mice compared to the sham-immunised mouse groups (Figure 2C). The significant differences were maintained when using different combinations of CpG and TGMP (Figure S8B). A mixed IgG1 and IgG2a isotype profile was observed for all the experimental conditions assayed (Figure 2C and Figure S8C).

The levels of parasite antigen-specific IgA were found to be significantly elevated at 3, 9, and 13 weeks following the TGMP+CpG booster immunisation in infected mice when compared to infected sham-immunised mice (Figure 2D). Using lower quantities of TGMP (10 µg) for immunisation also significantly increased IgA levels. However, when lower quantities of CpG (5 µg) were used, no differences were observed between TGMP+CpG-immunised animals and sham-immunised animals, indicating that the amount of adjuvant is a key factor in achieving significant specific mucosal immunity (Figure S8D). 

### 3.8. TGMP Immunisation Did Not Affect the Number of Total and Memory Splenic T Cells Detected After T. gondii Infection

T cells play a critical role in immunity against *T. gondii* infection, essential in developing protective immunity against this parasite [3]. Subsequent experiments enabled us to investigate the role of vaccination-induced T cells from the very earliest stages following infection. For this, mice were infected three weeks after the boost immunisation and sacrificed one day post-infection (1 dpi; Figure 3A). The memory phenotype of T cells was evaluated ex vivo by flow cytometry. No statistically significant differences were observed in the total numbers of CD4^+^ and CD8^+^ T cells (Figure 3B), or in the numbers of effector memory T cells (CD4^+^ or CD8^+^ TEM) and central memory T cells (CD4^+^ or CD8^+^ TCM) in both the TGMP+CpG- and CpG-immunised animals (Figure 3C,D).

### 3.9. IFN-γ Production Is Induced by TGMP Immunisation in Response to T. gondii Infection

IFN-γ is a crucial cytokine involved in the immune response against *T. gondii*, essential for controlling the parasite growth and ensuring host survival [22]. The levels of IFN-γ produced by splenocytes from immunised mice after recall stimulation with TGMP provided valuable information about the strength and specificity of the cellular immune response induced by TGMP+CpG or CpG immunisations. Mice were infected three weeks after the boost immunisation and sacrificed 1 dpi (Figure 3A).

A significantly higher production of IFN-γ by spleen cells of TGMP+CpG-immunised animals was observed in response to TGMP stimulation when compared with unstimulated cells (Figure 3E; Ctrl-; **** *p* < 0.0001). Additionally, a significant increase in IFN-γ production by TGMP-stimulated splenocytes from infected TGMP+CpG mice was observed, compared to infected CpG-immunised mice (### *p* < 0.001; Figure 3E). These findings indicate that TGMP+CpG immunisation induces a TGMP-specific Th1-type immunity. The ex vivo stimulation with TgS did not result in a significantly higher production of IFN-γ compared to unstimulated cells, and no significant differences were observed between the ability of splenocytes from *T. gondii*-infected TGMP+CpG- and CpG sham-immunised animals to produce IFN-γ in response to TgS (Figure 3E).

### 3.10. TGMP Immunisation Induces T Cell Activation in Response to T. gondii Infection

Total and activated spleen CD4^+^ and CD8^+^ T cells from animals immunised according to the same experimental design described previously (Figure 3A) were assessed by flow cytometry, following ex vivo stimulation with specific (TGMP and TgS) and non-specific (PMA/IONO) stimuli. No differences were observed in the numbers of total or activated CD4^+^ T cells and CD8^+^ T cells from infected TGMP+CpG-immunised animals and CpG sham-immunised animals (Figure 3F,G). However, after TGMP stimulation, significantly higher numbers of total and activated CD4^+^ and CD8^+^T cells producing IFN-γ^+^ were detected in the infected TGMP+CpG group compared to the infected sham-immunised group. A significant increase in the number of total and activated CD4^+^T cells producing IL-17 was also observed. Moreover, a substantial increase in the number of total and activated CD8^+^ T cells producing GranzB was observed following TGMP stimulation in the TGMP+CpG group compared to the sham-immunised control group (Figure 3F,G). These results altogether indicate that in infected mice of the TGMP+CpG-immunised mouse group, T cells are activated and produce protective cytokines in response to parasite antigens. 

## 4. Discussion

In this work, an i.n. immunisation approach based on a previously developed vaccination strategy against neosporosis was assessed [11,23,24]. The aim was to evaluate whether protection against i.p. infection with *T. gondii* could be achieved by employing an immunisation route capable of eliciting both systemic and mucosal antigen-specific responses. Infection was carried out intraperitoneally, as several studies have used i.p. infection with *T. gondii* Me49 tachyzoites to analyse the acute phase infection [25,26]. Studies using the *N. caninum* model have shown that this mucosal vaccination strategy protects not only against intragastric *N. caninum* infection but also against i.p. infection, a route that efficiently promotes rapid widespread parasite dissemination [11,24,27]. In this study, mice were challenged through the i.p. route. This is a limitation, since the natural route of *T. gondii* infection is oral, primarily through ingestion of tissue cysts or oocysts in contaminated food or water [28]. Nevertheless, previous studies from our group using an experimental *N. caninum* infection model demonstrated that, via both intragastric and i.p. routes of infection, significant protection was observed in intranasally immunised mice with NcMP plus CpG, along with a measurable immune response to infection [11,24,27]. Based on these observations, the i.p. route was selected in the present study because it provides a more controlled and reproducible route of infection, reducing technical variability and minimising animal distress associated with the oral gavage procedure. In this work, TGMP was used as a target antigen in combination with CpG adjuvant, a TLR9 agonist that promotes Th1-type immunity mediated by IFN-γ, the prototypical host-protective response in toxoplasmosis [29]. The use of CpG as an adjuvant leads to robust mucosal and systemic immunity [11,30], which is important for preventing and controlling toxoplasmosis [31]. i.n. vaccination promoted mucosal immunity, assessed by the high levels of anti-TGMP secretory IgA, which may agglutinate and/or neutralise parasites at the intestinal surface and prevent their invasion of enterocytes, as observed for anti-NcMP specific IgA [11]. Other studies have reported that i.n. *T. gondii* vaccines elicited strong intestinal IgA responses associated with significantly reduced pathology [32,33]. TGMP extract was enriched in proteins with molecular weights of approximately 19, 30, and 35 kDa. This protein profile differed from that of similarly produced NcMP, reflecting genetic divergence and functional specialisation, leading to unique antigenic signatures and immune responses in each parasite [11,34]. Here, *T. gondii* GRA2, GRA7, SRS25, SRS29B, SRS34A, toxofilin, NAC-domain containing protein, NAC subunit beta, and MIC-10 were identified as TGMP immunodominant proteins. The GRA2 (28 kDa) and GRA7 (29 kDa) proteins are crucial for parasite replication and immune modulation, with GRA7 being expressed in all parasite stages and highly targeted in vaccine studies [20,35]. Furthermore, three surface antigens with a score of 8.0 were identified as belonging to the SRS protein superfamily (sequences related to SAG1 proteins): SRS25, SRS29B (formerly known as SAG1), and SRS34A (formerly known as SAG2A). SRS25, SRS29B, and SRS34A are surface antigens belonging to the SRS protein superfamily, which are involved in host immune modulation and virulence. SRS25 is expressed in both tachyzoites and sporozoites, and while it has a “degraded” SRS domain, it still modulates the host immune response [36]. SRS29B and SRS34A are critical for host-parasite interactions, mediating invasion and immune evasion [37]. Toxofilin is an actin-binding protein found in *T. gondii* tachyzoites, which may facilitate host cell invasion by disrupting the actin network [38]. MIC10, an 18 kDa protein, is highly expressed in tachyzoites and involved in protein trafficking and targeting [39]. NAC-domain-containing protein (20.5 kDa) and the NAC subunit beta (38.8 kDa) are associated with protein trafficking and targeting during expression [40]. The diverse functions, structures, and localisations of these TGMP immunogenic proteins make them strong candidates for inducing both humoral and cellular immunity upon immunisation.

DCs are present in the nasal-associated lymphoid tissue and are interconnected with epithelial cells, M cells, subepithelial B cells, and CD4^+^ and CD8^+^ T cells, and are pivotal in the protection against *T. gondii* infection [41]. Thus, the immunogenicity of TGMP and TgS was evaluated in vitro using murine BMDCs (characterised as CD11c^+^F4/80^−^Ly6G^−^) as a model. The use of primary cells closely reflects the in vivo activation of DCs. In our experimental setup, exposure to TGMP and TgS significantly increased the expression of MHC II and CD86 on DCs, indicating that these antigen extracts, per se, enhance DC activation. Although the specific Pattern Recognition Receptors activated by TGMP were not identified in the present study, previous reports suggest that *T. gondii* membrane- and surface-associated components may interact with Toll-like receptors, including TLR2 and TLR4, leading to DC activation and pro-inflammatory cytokine production. In particular, *T. gondii* glycosylphosphatidylinositols (GPIs) have been shown to activate both TLR2 and TLR4 signalling pathways [42,43]. The precise receptors and signalling pathways involved remain to be elucidated. Our results also suggest a preferential increase in the percentage of dividing primed CD4^+^ T cells, compared to CD8^+^ T cells, when co-cultured with BMDC previously stimulated with TGMP or TgS. This observation is consistent with the enhanced expression of MHC II and CD86 detected in BMDCs stimulated with TGMP and TgS. Exogenous antigens are preferentially processed and presented via the MHC II pathway, favouring CD4^+^ T cell activation, whereas CD8^+^ T cell activation generally depends on efficient cross-presentation. In the context of *T. gondii*, DCs are recognised as central mediators of CD4^+^ T cell priming and cytokine-driven immunity [44,45].

Another essential aspect of the immune response to *T. gondii* infection is the production of parasite-specific antibodies [46]. Parasite-specific antibodies block parasite invasion of host cells, facilitate phagocytosis through opsonisation, and activate the classical complement pathway [47]. The immunisation strategy used here effectively induced serum TGMP-specific IgG1 and IgG2a, as well as intestinal TGMP-specific IgA. The use of 10 µg and 30 µg of TGMP in the immunising preparation induced similar IgG1 and IgG2a titres. However, mucosal immunity depended on the CpG dose, since reducing it from 10 µg to 5 µg failed to induce TGMP-specific IgA above control levels, as detected after infection. This finding is in accordance with other studies demonstrating that CpG effectively promotes IgA responses and antigen-specific IgG production by activating B cells [48]. The CpG adjuvant used in our formulation is a well-established TLR9 agonist that promotes dendritic cell maturation, IL-12 production, and Th1 polarisation [49,50,51]. Therefore, it is plausible that the activation of DCs results from a combined effect of CpG-mediated TLR9 stimulation and TGMP-mediated TLR2/TLR4 stimulation. TLR9 activation in DCs and B cells promotes cytokine production and creates a mucosal microenvironment favourable for IgA class-switch recombination and plasma cell differentiation [49,51,52]. The IgG2a/IgG1 ratio indicates that immunisation elicited a balanced Th1/Th2 response [53,54], which is considered important for the development of *T. gondii* resistance and host survival [55,56]. This is particularly significant given that BALB/c mice are naturally biased toward Th2-type immunity [57].

In the present study, we aimed to assess systemic vaccine efficacy rather than mucosal-mediated protection and to focus on the host’s response when the parasite overcomes the mucosal barrier. Previous *N. caninum* vaccination using this strategy demonstrated mucosal protection after intragastric infection and systemic protection following infection via the i.p. route. Based on our previous findings on *N. caninum* vaccination [24], in which the formulation combining membrane proteins with CpG elicited the strongest protective effect, while membrane proteins alone induced a comparatively weaker immune response and protection, a TGMP-only group was not included in the present study. The i.p. route ensures strict dose control, high reproducibility, and straightforward monitoring, while the peritoneal cavity serves as the primary site for parasite multiplication and recruitment of neutrophils, lymphocytes, and monocytes, facilitating evaluation of broad systemic immunity [58].

A protective effect induced by immunisation was detected by assessing parasite load in PE, spleen, and lungs, but not consistently in other organs analysed, such as the liver, kidneys, heart, and brain. This data indicates that the liver, kidneys, heart, and brain exhibited lower parasite loads during the acute infection period.

Monocytes, neutrophils, and DCs play a key role in protecting the host against *T. gondii* by producing IL-12, which activates NK cells and T cells to secrete IFN-γ [3]. CD4^+^ T cells are a significant source of IFN-γ during both acute and chronic infections. This cytokine is crucial for killing and inhibiting parasite replication [3,46]. CD8^+^ T cells, which also produce IFN-γ, are important effector cells for *T. gondii* control through cytotoxic activity [3]. Our results demonstrate that immunisation with TGMP+CpG, unlike CpG alone, induces activation of both CD4^+^ and CD8^+^ T cells and elicits a significant TGMP-specific IFN-γ-mediated response.

IL-17 is another important cytokine in the control of *T. gondii* infection. Indeed, it facilitates neutrophil recruitment, mucosal defence, and macrophage activation, key factors in controlling pathogens such as *T. gondii* [59]. i.n. immunisation with TGMP+CpG triggered TGMP-specific T cell responses, characterised by increased CD4^+^ T cells producing IFN-γ and IL-17, and CD8^+^ T cells producing Granzyme B and IFN-γ, indicating a protective immune response. The activation of CD4^+^ T cells towards IL-17 and IFN-γ production reflects a mixed Th1/Th17 helper T cell response [60,61]. Although Th17 cells are often associated with hyperinflammation [62], their plasticity allows them to acquire IFN-γ production after vaccination, thereby enhancing Th1-type effector responses and promoting pathogen elimination, albeit at a potential cost of increased inflammation [63,64].

In *T. gondii* infection, Th17 responses are linked to both host defence and immunopathology, whereas durable control of the parasite relies predominantly on IFN-γ-driven Th1 immunity; chronic infection and some vaccine or immunisation settings favour Th1 polarisation and IFN-γ production, which promote parasite elimination but can also contribute to inflammation [65,66]. This dual-axis response (Th1/Th17) mirrors successful vaccine strategies against *T. gondii* and other intracellular pathogens, such as influenza and *Listeria*, where coordinated cytotoxicity and cytokine signalling are essential for effective protection [67,68,69,70].

The production of Granz B by CD8^+^ T cells is particularly important, as this key effector molecule enables cytotoxic T lymphocytes (CTLs) to directly eliminate infected host cells [67]. Granz B production indicates cytotoxic potential of CD8^+^ T cells to directly eliminate target cells, thereby contributing to the effectiveness of vaccine-induced immunity [67]. The production of IFN-γ by CD8^+^ T cells additionally contributes to activating macrophages, enhancing their ability to clear pathogens and regulating the contraction and memory profile of CD8^+^ T cells, which is critical for establishing long-term immunity [68]. IFN-γ also suppresses IL-17 production by CD8^+^ T cells, ensuring that these cells maintain a focused cytotoxic and Th1-type response rather than adopting Th17-like characteristics [68,71]. The absence of IL-17 production in CD8^+^ T cells in this context is not a concern, as it instead indicates the specialisation of these cells for cytotoxic and Th1-type immunity [63,68].

Although the TGMP+CpG formulation induced Th1/Th17-associated responses and CD8+ T cell activation, including IFN-γ and Granzyme B production, the observed protection remained partial. This finding is consistent with previous studies showing that, although IFN-γ-mediated cellular immunity is essential for controlling *T. gondii* infection, it is not always sufficient to achieve complete protection, particularly against highly virulent tachyzoite challenge strains [72,73]. Several factors may contribute to this partial protection. First, *T. gondii* possesses multiple immune evasion mechanisms that allow rapid intracellular replication and dissemination [73,74]. Second, protection against infection likely requires a highly coordinated immune response involving innate immune activation, adequate CD4^+^ T cell help, IFN-γ-producing CD4^+^ and CD8^+^ T cells, long-lived memory cells, and efficient mucosal immunity [72,75].

In this study, the total *T. gondii* extract, TgS, was used as an internal control because it contains nearly all *T. gondii* proteins, including soluble proteins, and has been shown to confer partial protection against *T. gondii* infection [76,77]. TgS exhibited the same ability as TGMP to activate DCs and stimulate antigen-specific T-cell proliferation. However, TgS did not increase IFN-γ production by spleen cells compared with unstimulated controls, and no difference in IFN-γ levels was observed between the two groups of animals (CpG- and CpG+TGMP-immunised). In addition, TgS stimulation increased the number of CD4^+^ T lymphocytes producing IFN-γ or IL-17, similar to TGMP, but had no effect on CD8^+^ T cells, unlike TGMP. These observations suggest that TGMP antigens could exert greater protective activity than those of TgS, as it induces CD8^+^ T cell responses, specifically the production of Granzyme B and IFN-γ.

Overall, the T cell responses induced in TGMP+CpG-immunised mice reveal a multifaceted defence that combines CD8+ T cell cytotoxicity and CD4^+^ T cell cytokine support, both of which are essential for establishing durable protection against *T. gondii* infection.

Our study shows that TGMPs are a valuable vaccine target, inducing antibody and cell-mediated immunity that confer partial protection against acute murine *T. gondii* infection.

The next step will be to explore a multi-antigen approach, such as chimeric constructs combining immunodominant domains from the nine immunogenic proteins identified in TGMP. This will allow us to simplify delivery and boost immunogenicity, a determinant of vaccine efficacy, thereby offering considerable protection against this widespread and potentially fatal parasitic infection.

## 5. Conclusions

This study demonstrates that i.n. immunisation with a vaccine formulation comprising TGMP and a CpG adjuvant induces both systemic and mucosal immune responses, which are associated with partial protection against acute toxoplasmosis. This study identified several immunogenic TGMP-associated proteins, including GRA2, GRA7, SRS family proteins, toxofilin, and MIC10, highlighting their potential as vaccine targets.

TGMP+CpG immunisation promoted parasite-specific IgG and mucosal IgA production, associated with a reduction in parasite burden. It further elicited antigen-specific CD4^+^ T cells producing IFN-γ and IL-17, and CD8^+^ T cells producing IFN-γ and Granz B.

Although protection remained partial, the results support the potential of TGMP-based i.n. vaccination strategies to elicit multifaceted immune responses against *T. gondii*. These findings strongly support the development of multi-antigen vaccine strategies, which are likely to enhance protective efficacy, representing a promising path toward an effective vaccine against toxoplasmosis.

## Figures and Tables

**Table 1:** Description of the proteins identified by mass spectrometry.

Protein	Sequence Coverage (%)	N° of Unique Peptides	Score Sequest HT	Molecular Weight (kDa) ^a^	Accession Number
Dense granule protein 7	45	10	21.56	25.8	O00933
SAG-related sequence SRS25	34	3	9.17	20.8	S8FBZ9
Dense granule protein 2	28	6	15.47	19.8	P13404
SAG-related sequence SRS34A	26	3	5.83	19.1	A0A125YIJ3
Toxofilin	19	4	5.74	26.8	S8G4J8
SAG-related sequence SRS29B	15	4	8.4	34.7	A0A125YP09
Nascent polypeptide-associated complex (NAC) domain-containing protein	15	2	5.22	20.5	A0A125YY47
Nascent polypeptide-associated complex subunit beta	14	4	9.07	38.8	S8GBN3
Microneme protein MIC10	11	2	4.76	23.1	A0A125YLT9

The UniProt 2021_03 database was considered for the taxonomic selection of the proteome of *T. gondii* (strain ATCC 50,611/Me49). Protein identification was performed with Proteome Discoverer software (v2.5, Thermo Scientific). The proteins presented were identified by mass spectrometry after an approach of immunoproteomics and were considered based on the sequence coverage ≥0%, unique peptide ≥2, and a Sequest HT score ≥1.5. ^a^ Information obtained from the UniProt database for the specified entry. The sequence coverage in %, the number of unique peptides, the Sequest HT score, the molecular weight in kDa, and the accession number in Uniprot are presented for each protein.

## Data Availability

The data presented in this study are available on reasonable request from the corresponding author.

## Supplementary Materials

The following supporting information can be downloaded at: https://www.mdpi.com/article/10.3390/vaccines14060539/s1, Figure S1. Gating strategy used in the analysis of activation marker expression (MHCII, CD80 and CD86) on bone-marrow derived dendritic cells (BMDC). Figure S2. Gating strategy used for the quantification of CD4 and CD8 T cell proliferation in response to TGMP-loaded DCs. Figure S3. Gating strategy used for the analysis of spleen memory phenotype of CD4^+^ and CD8^+^ T cells. Figure S4. Gating strategy used for the evaluation of spleen T cell activation and responses to TGMP-specific stimulus. Figure S5. Effect of TGMP and TgS supplemented with Polymyxin B (P) on bone marrow-derived dendritic cell activation markers MHCII and CD86 after 4 and 16 hours of stimulation. Figure S6. Dose-ranging evaluation of CpG adjuvant and TGMP in intranasal vaccination: impact on mucosal and systemic immunity. Figure S7. Raw data underlying the analyses presented in Figure 1H and Figure S5D. Figure S8. Dose-ranging evaluation of CpG adjuvant and TGMP in intranasal vaccination: impact on protection against acute infection and TGMP-specific humoral responses. File S1: Proteomic Data Acquisition and Identification Using Tune 2.11 and Proteome Discoverer 2.5 Against the UniProt 2021_03 *T. gondii* Me49 Database. Table S1: Animal groups used in this study and corresponding experiments. Table S2: Animal weight monitoring after infection.
