# Supplementary material for: Protective Effect Against Acute Experimental Toxoplasmosis Conferred by Intranasal Immunisation with Toxoplasma gondii Membrane Proteins Plus CpG Adjuvant
Source: Vaccines (Basel). 2026 Jun 17;14(6):539. doi: 10.3390/vaccines14060539 (PMC13308317; doi:10.3390/vaccines14060539)
Supplement: Supplementary file 1 [file vaccines-14-00539-s001.zip › Figure S1.pptx]

## Slide 1
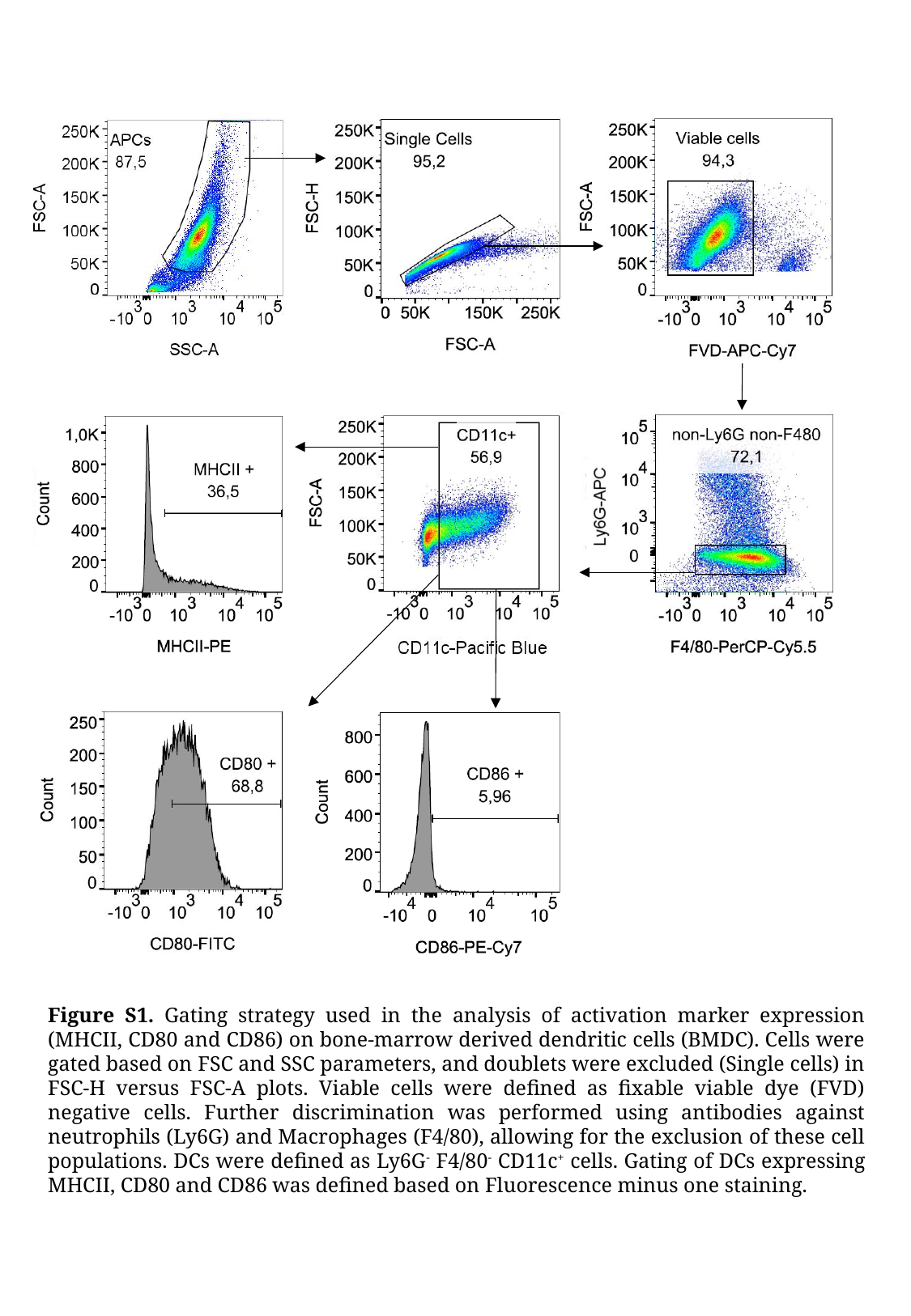

Figure S1. Gating strategy used in the analysis of activation marker expression (MHCII, CD80 and CD86) on bone-marrow derived dendritic cells (BMDC). Cells were gated based on FSC and SSC parameters, and doublets were excluded (Single cells) in FSC-H versus FSC-A plots. Viable cells were defined as fixable viable dye (FVD) negative cells. Further discrimination was performed using antibodies against neutrophils (Ly6G) and Macrophages (F4/80), allowing for the exclusion of these cell populations. DCs were defined as Ly6G- F4/80- CD11c+ cells. Gating of DCs expressing MHCII, CD80 and CD86 was defined based on Fluorescence minus one staining.
