# Supplementary material for: Protective Effect Against Acute Experimental Toxoplasmosis Conferred by Intranasal Immunisation with Toxoplasma gondii Membrane Proteins Plus CpG Adjuvant
Source: Vaccines (Basel). 2026 Jun 17;14(6):539. doi: 10.3390/vaccines14060539 (PMC13308317; doi:10.3390/vaccines14060539)
Supplement: Supplementary file 1 [file vaccines-14-00539-s001.zip › Figure S2.pptx]

## Slide 1
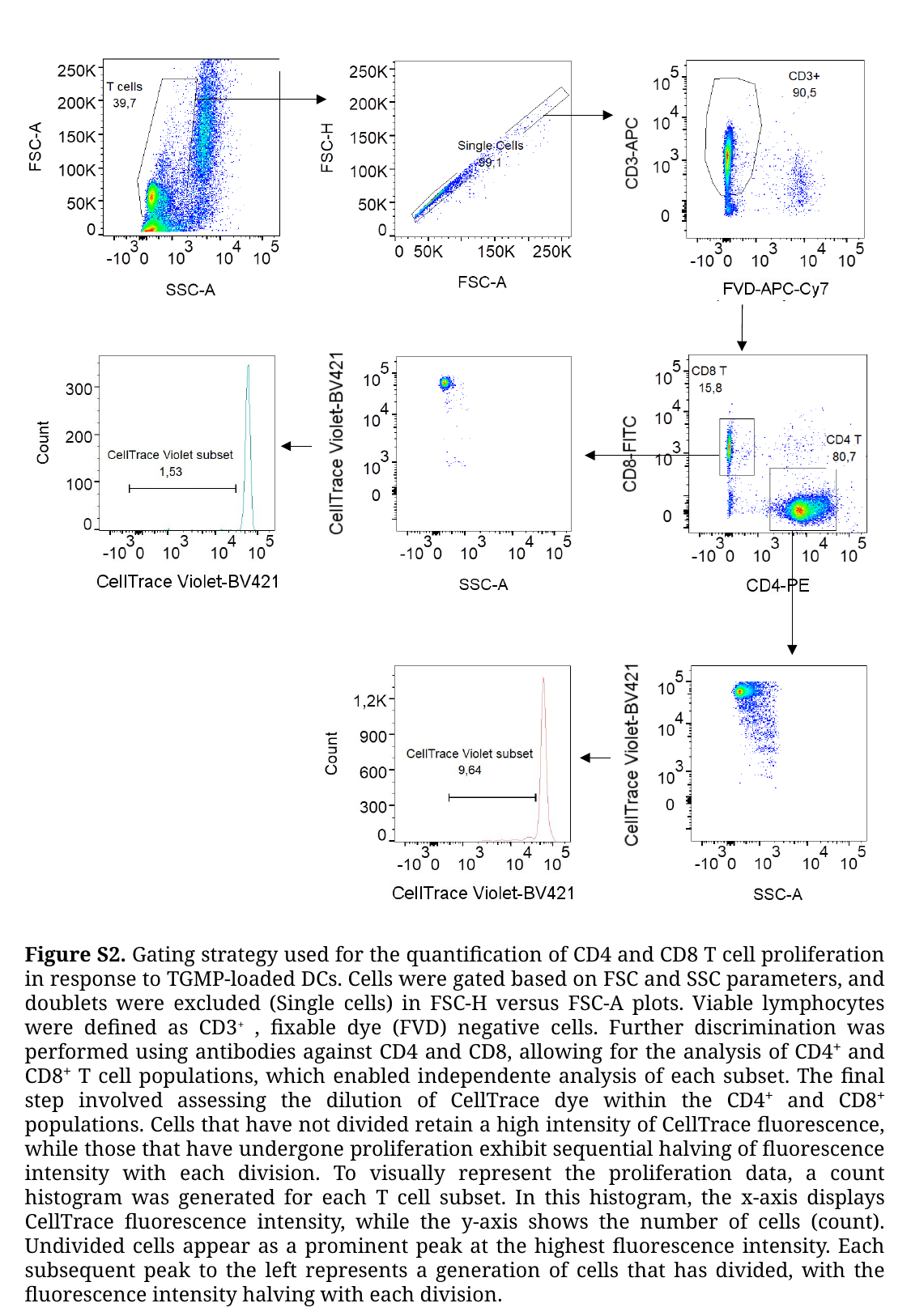

Figure S2. Gating strategy used for the quantification of CD4 and CD8 T cell proliferation in response to TGMP-loaded DCs. Cells were gated based on FSC and SSC parameters, and doublets were excluded (Single cells) in FSC-H versus FSC-A plots. Viable lymphocytes were defined as CD3+ , fixable dye (FVD) negative cells. Further discrimination was performed using antibodies against CD4 and CD8, allowing for the analysis of CD4⁺ and CD8⁺ T cell populations, which enabled independente analysis of each subset. The final step involved assessing the dilution of CellTrace dye within the CD4⁺ and CD8⁺ populations. Cells that have not divided retain a high intensity of CellTrace fluorescence, while those that have undergone proliferation exhibit sequential halving of fluorescence intensity with each division. To visually represent the proliferation data, a count histogram was generated for each T cell subset. In this histogram, the x-axis displays CellTrace fluorescence intensity, while the y-axis shows the number of cells (count). Undivided cells appear as a prominent peak at the highest fluorescence intensity. Each subsequent peak to the left represents a generation of cells that has divided, with the fluorescence intensity halving with each division.
