# Supplementary material for: Protective Effect Against Acute Experimental Toxoplasmosis Conferred by Intranasal Immunisation with Toxoplasma gondii Membrane Proteins Plus CpG Adjuvant
Source: Vaccines (Basel). 2026 Jun 17;14(6):539. doi: 10.3390/vaccines14060539 (PMC13308317; doi:10.3390/vaccines14060539)
Supplement: Supplementary file 1 [file vaccines-14-00539-s001.zip › Figure S3.pptx]

## Slide 1
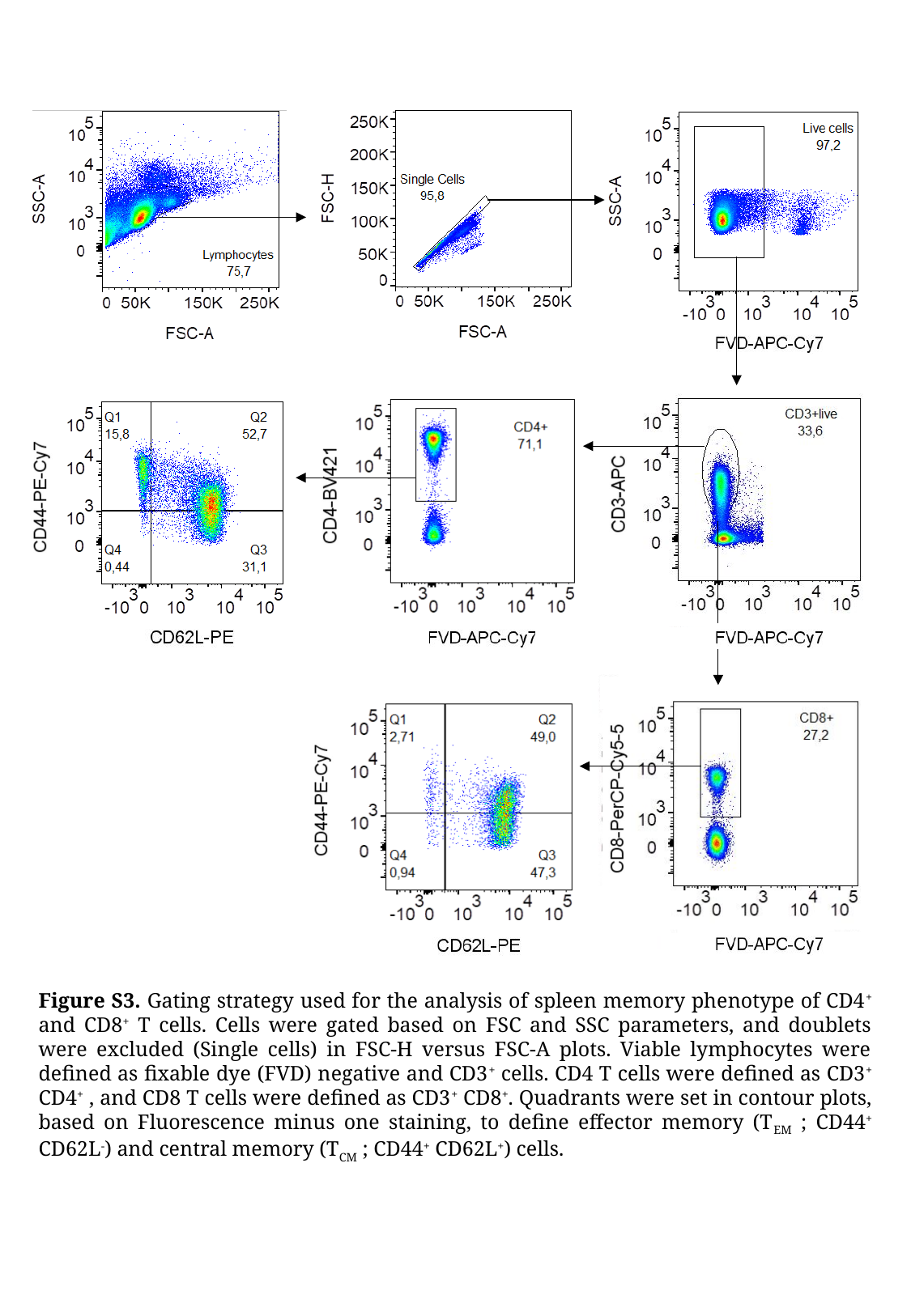

Figure S3. Gating strategy used for the analysis of spleen memory phenotype of CD4+ and CD8+ T cells. Cells were gated based on FSC and SSC parameters, and doublets were excluded (Single cells) in FSC-H versus FSC-A plots. Viable lymphocytes were defined as fixable dye (FVD) negative and CD3+ cells. CD4 T cells were defined as CD3+ CD4+ , and CD8 T cells were defined as CD3+ CD8+. Quadrants were set in contour plots, based on Fluorescence minus one staining, to define effector memory (TEM ; CD44+ CD62L-) and central memory (TCM ; CD44+ CD62L+) cells.
