# Supplementary material for: Protective Effect Against Acute Experimental Toxoplasmosis Conferred by Intranasal Immunisation with Toxoplasma gondii Membrane Proteins Plus CpG Adjuvant
Source: Vaccines (Basel). 2026 Jun 17;14(6):539. doi: 10.3390/vaccines14060539 (PMC13308317; doi:10.3390/vaccines14060539)
Supplement: Supplementary file 1 [file vaccines-14-00539-s001.zip › Figure S4.pptx]

## Slide 1
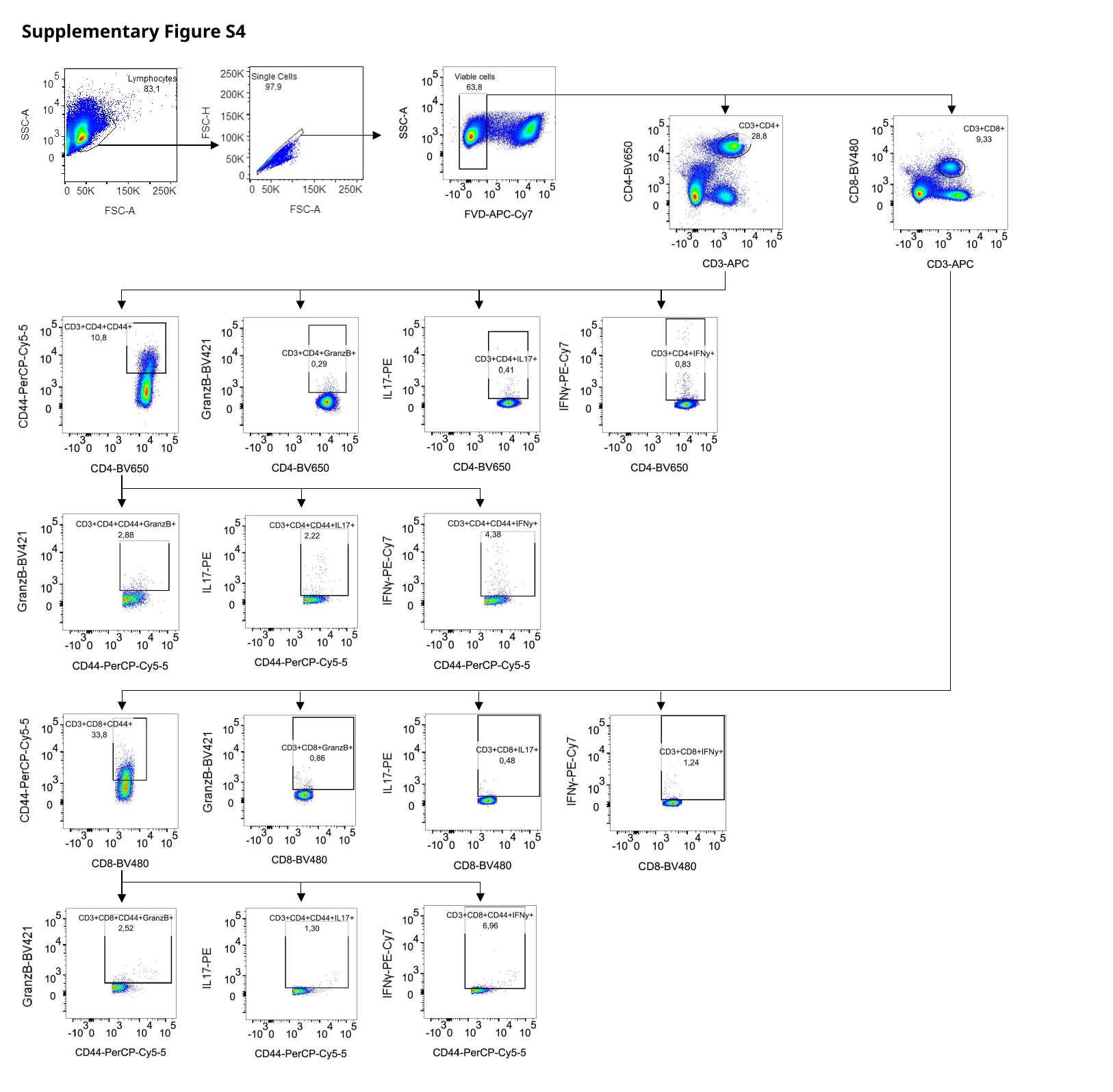

Supplementary Figure S4

## Slide 2
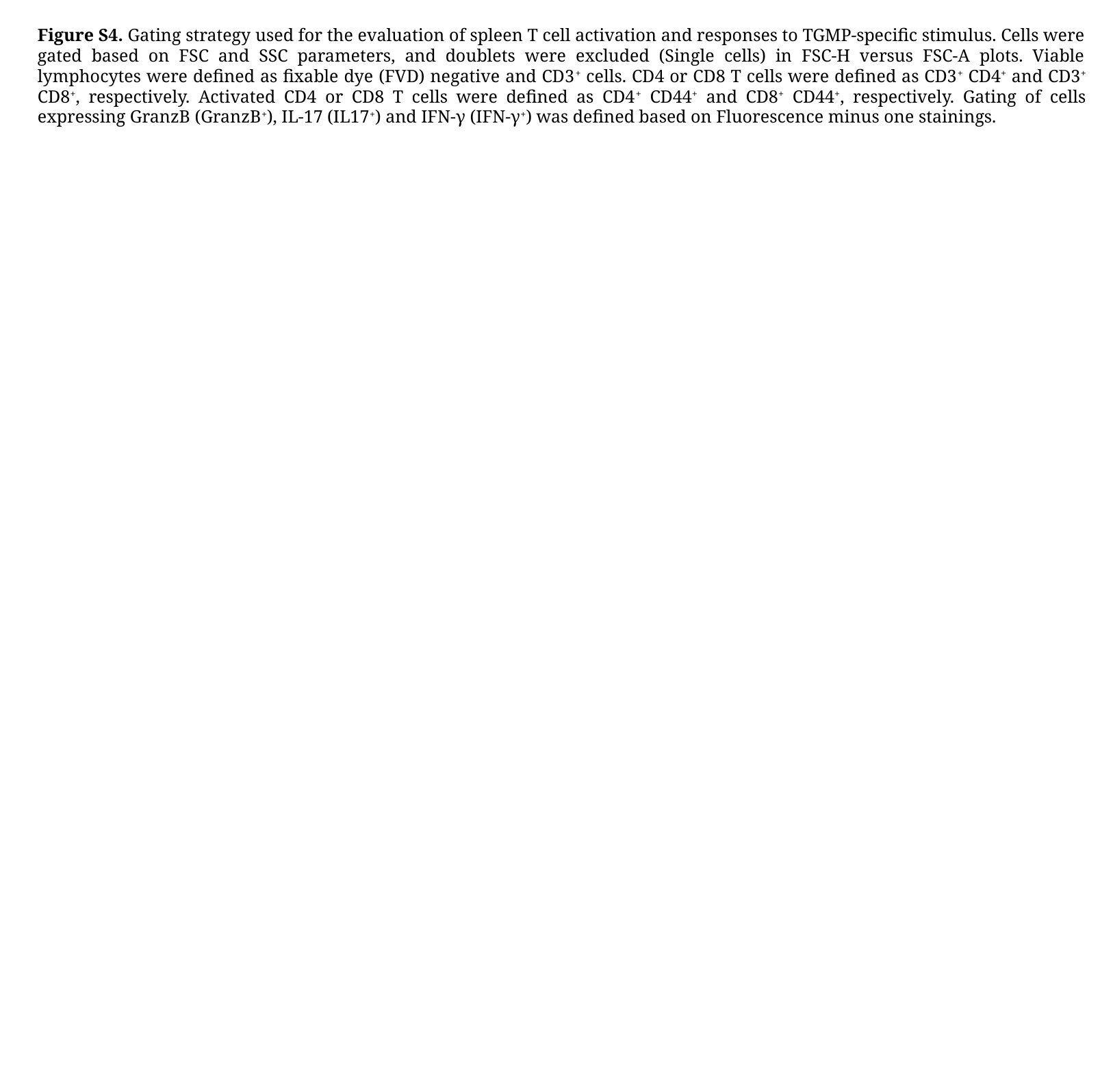

Figure S4. Gating strategy used for the evaluation of spleen T cell activation and responses to TGMP-specific stimulus. Cells were gated based on FSC and SSC parameters, and doublets were excluded (Single cells) in FSC-H versus FSC-A plots. Viable lymphocytes were defined as fixable dye (FVD) negative and CD3+ cells. CD4 or CD8 T cells were defined as CD3+ CD4+ and CD3+ CD8+, respectively. Activated CD4 or CD8 T cells were defined as CD4+ CD44+ and CD8+ CD44+, respectively. Gating of cells expressing GranzB (GranzB+), IL-17 (IL17+) and IFN-γ (IFN-γ+) was defined based on Fluorescence minus one stainings.
