# Supplementary material for: Protective Effect Against Acute Experimental Toxoplasmosis Conferred by Intranasal Immunisation with Toxoplasma gondii Membrane Proteins Plus CpG Adjuvant
Source: Vaccines (Basel). 2026 Jun 17;14(6):539. doi: 10.3390/vaccines14060539 (PMC13308317; doi:10.3390/vaccines14060539)
Supplement: Supplementary file 1 [file vaccines-14-00539-s001.zip › Figure S5.pptx]

## Slide 1
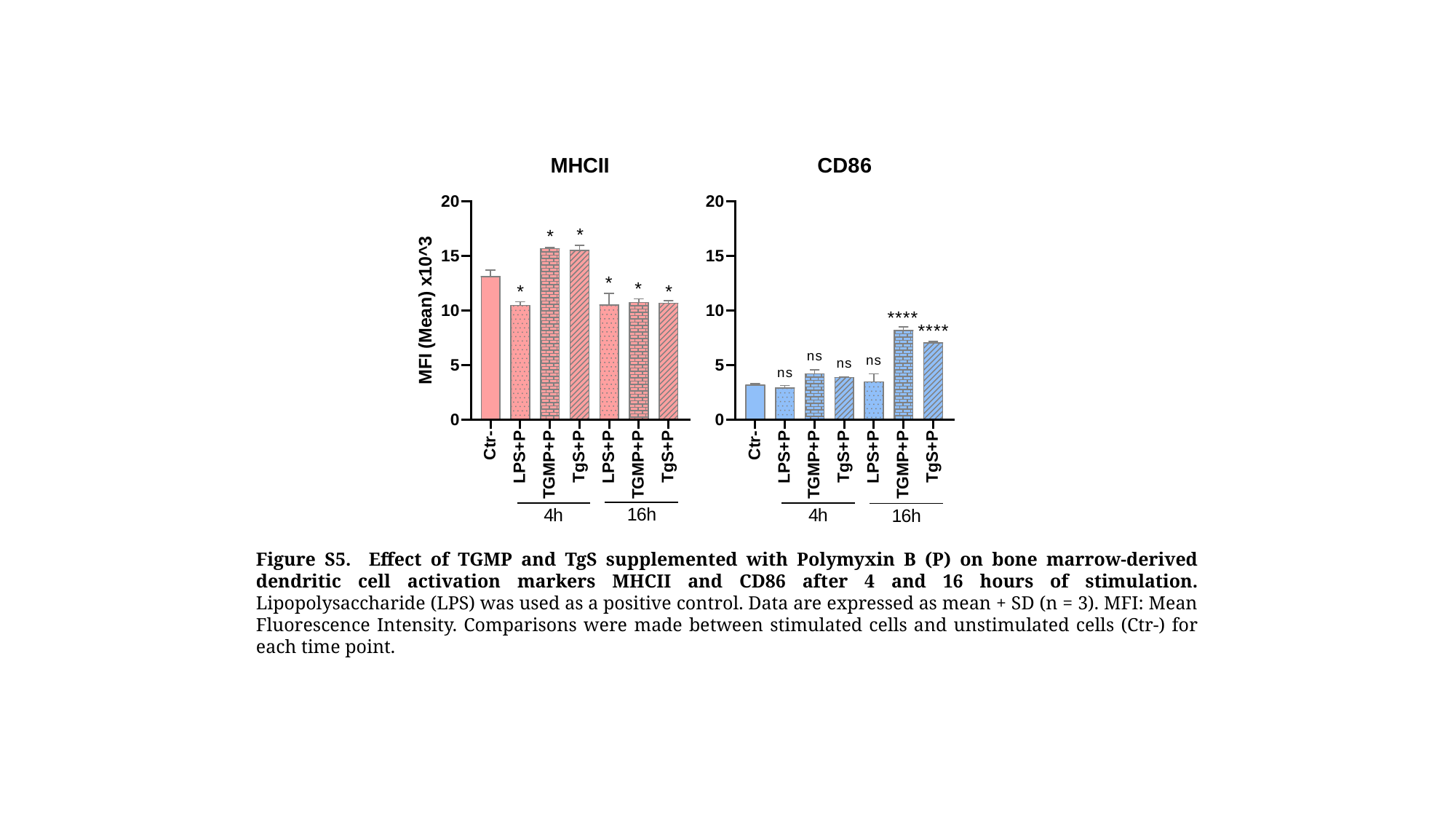

Figure S5. Effect of TGMP and TgS supplemented with Polymyxin B (P) on bone marrow-derived dendritic cell activation markers MHCII and CD86 after 4 and 16 hours of stimulation. Lipopolysaccharide (LPS) was used as a positive control. Data are expressed as mean + SD (n = 3). MFI: Mean Fluorescence Intensity. Comparisons were made between stimulated cells and unstimulated cells (Ctr-) for each time point.
