# Supplementary material for: Protective Effect Against Acute Experimental Toxoplasmosis Conferred by Intranasal Immunisation with Toxoplasma gondii Membrane Proteins Plus CpG Adjuvant
Source: Vaccines (Basel). 2026 Jun 17;14(6):539. doi: 10.3390/vaccines14060539 (PMC13308317; doi:10.3390/vaccines14060539)
Supplement: Supplementary file 1 [file vaccines-14-00539-s001.zip › Figure S6.pptx]

## Slide 1
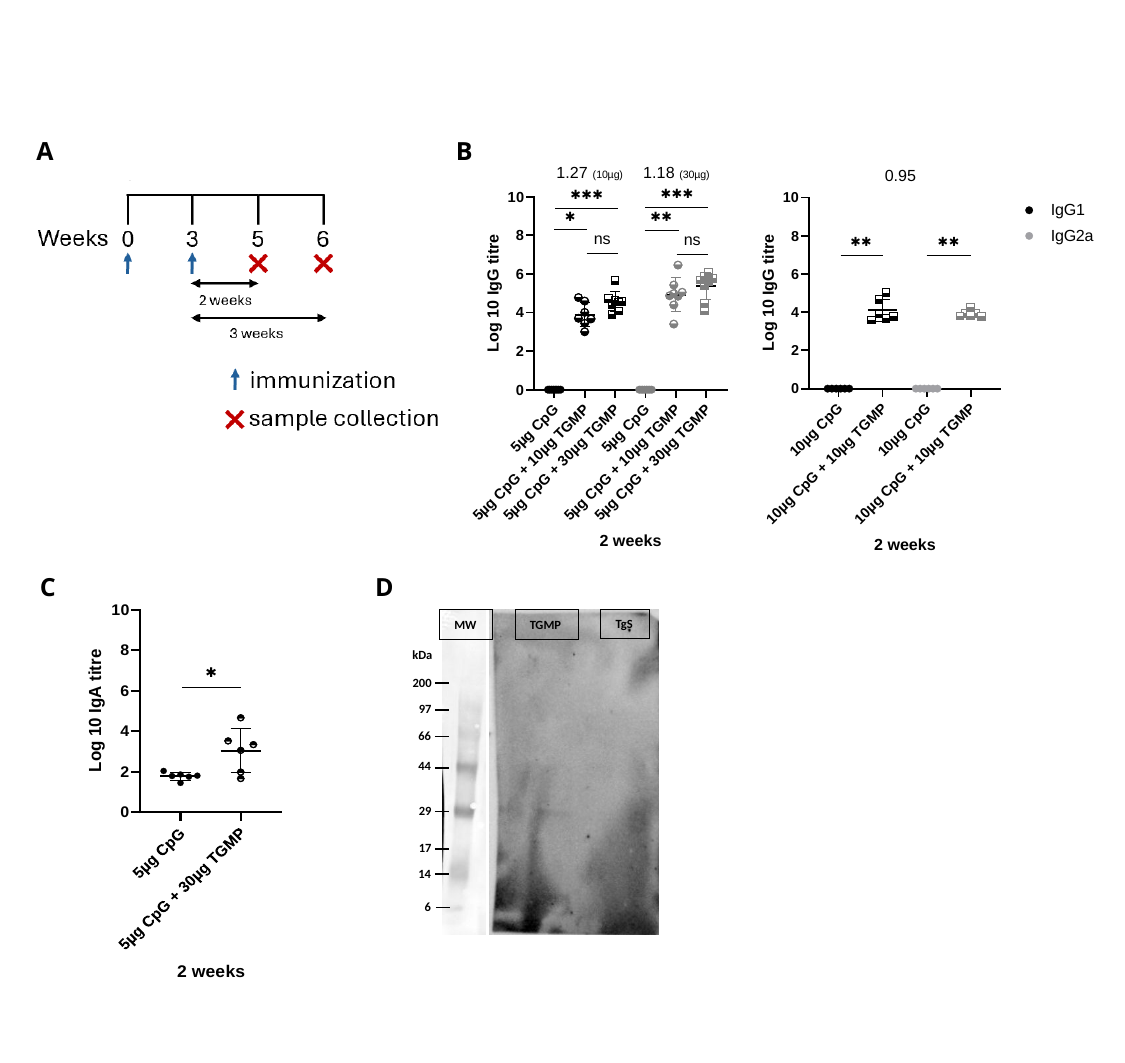

A
B
D
C
TgS
MW
TGMP
200
97
66
44
29
17
14
6
kDa

## Slide 2
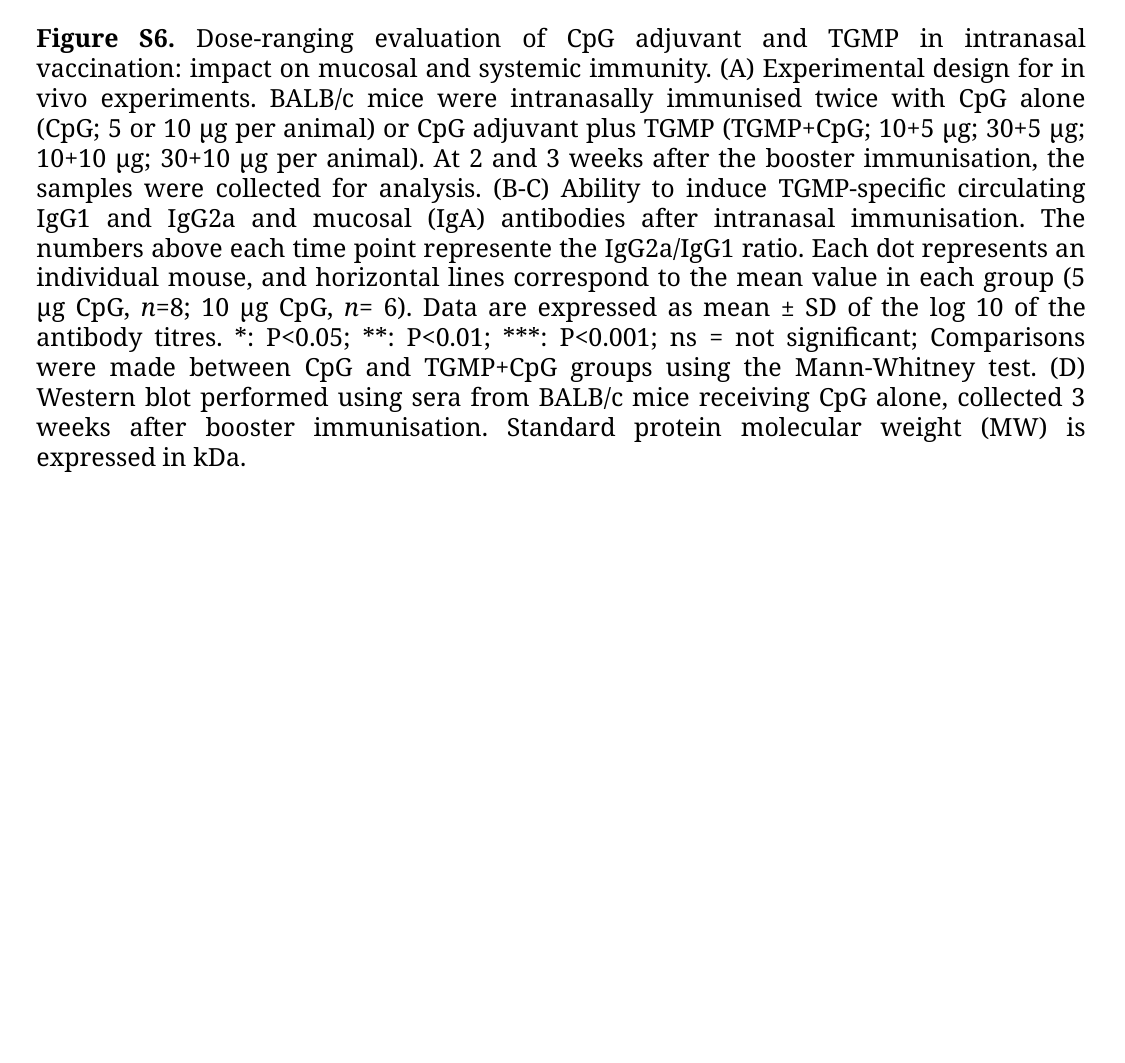

Figure S6. Dose-ranging evaluation of CpG adjuvant and TGMP in intranasal vaccination: impact on mucosal and systemic immunity. (A) Experimental design for in vivo experiments. BALB/c mice were intranasally immunised twice with CpG alone (CpG; 5 or 10 µg per animal) or CpG adjuvant plus TGMP (TGMP+CpG; 10+5 µg; 30+5 µg; 10+10 µg; 30+10 µg per animal). At 2 and 3 weeks after the booster immunisation, the samples were collected for analysis. (B-C) Ability to induce TGMP-specific circulating IgG1 and IgG2a and mucosal (IgA) antibodies after intranasal immunisation. The numbers above each time point represente the IgG2a/IgG1 ratio. Each dot represents an individual mouse, and horizontal lines correspond to the mean value in each group (5 µg CpG, n=8; 10 µg CpG, n= 6). Data are expressed as mean ± SD of the log 10 of the antibody titres. *: P<0.05; **: P<0.01; ***: P<0.001; ns = not significant; Comparisons were made between CpG and TGMP+CpG groups using the Mann-Whitney test. (D) Western blot performed using sera from BALB/c mice receiving CpG alone, collected 3 weeks after booster immunisation. Standard protein molecular weight (MW) is expressed in kDa.
