# Supplementary material for: Protective Effect Against Acute Experimental Toxoplasmosis Conferred by Intranasal Immunisation with Toxoplasma gondii Membrane Proteins Plus CpG Adjuvant
Source: Vaccines (Basel). 2026 Jun 17;14(6):539. doi: 10.3390/vaccines14060539 (PMC13308317; doi:10.3390/vaccines14060539)
Supplement: Supplementary file 1 [file vaccines-14-00539-s001.zip › Figure S7.pptx]

## Slide 1
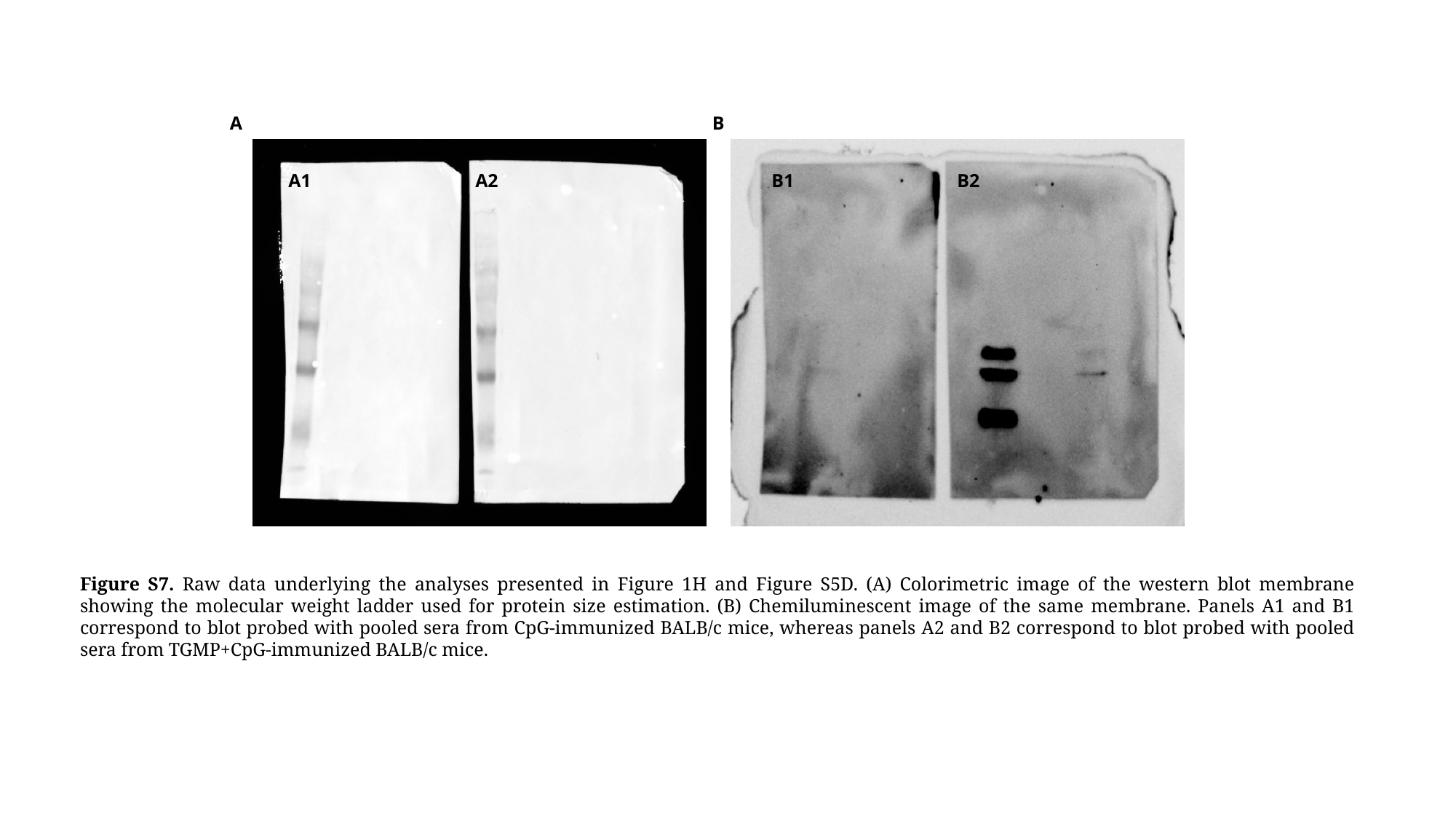

A
B
B2
B1
A1
A2
Figure S7. Raw data underlying the analyses presented in Figure 1H and Figure S5D. (A) Colorimetric image of the western blot membrane showing the molecular weight ladder used for protein size estimation. (B) Chemiluminescent image of the same membrane. Panels A1 and B1 correspond to blot probed with pooled sera from CpG-immunized BALB/c mice, whereas panels A2 and B2 correspond to blot probed with pooled sera from TGMP+CpG-immunized BALB/c mice.
