# Supplementary material for: Protective Effect Against Acute Experimental Toxoplasmosis Conferred by Intranasal Immunisation with Toxoplasma gondii Membrane Proteins Plus CpG Adjuvant
Source: Vaccines (Basel). 2026 Jun 17;14(6):539. doi: 10.3390/vaccines14060539 (PMC13308317; doi:10.3390/vaccines14060539)
Supplement: Supplementary file 1 [file vaccines-14-00539-s001.zip › Figure S8.pptx]

## Slide 1
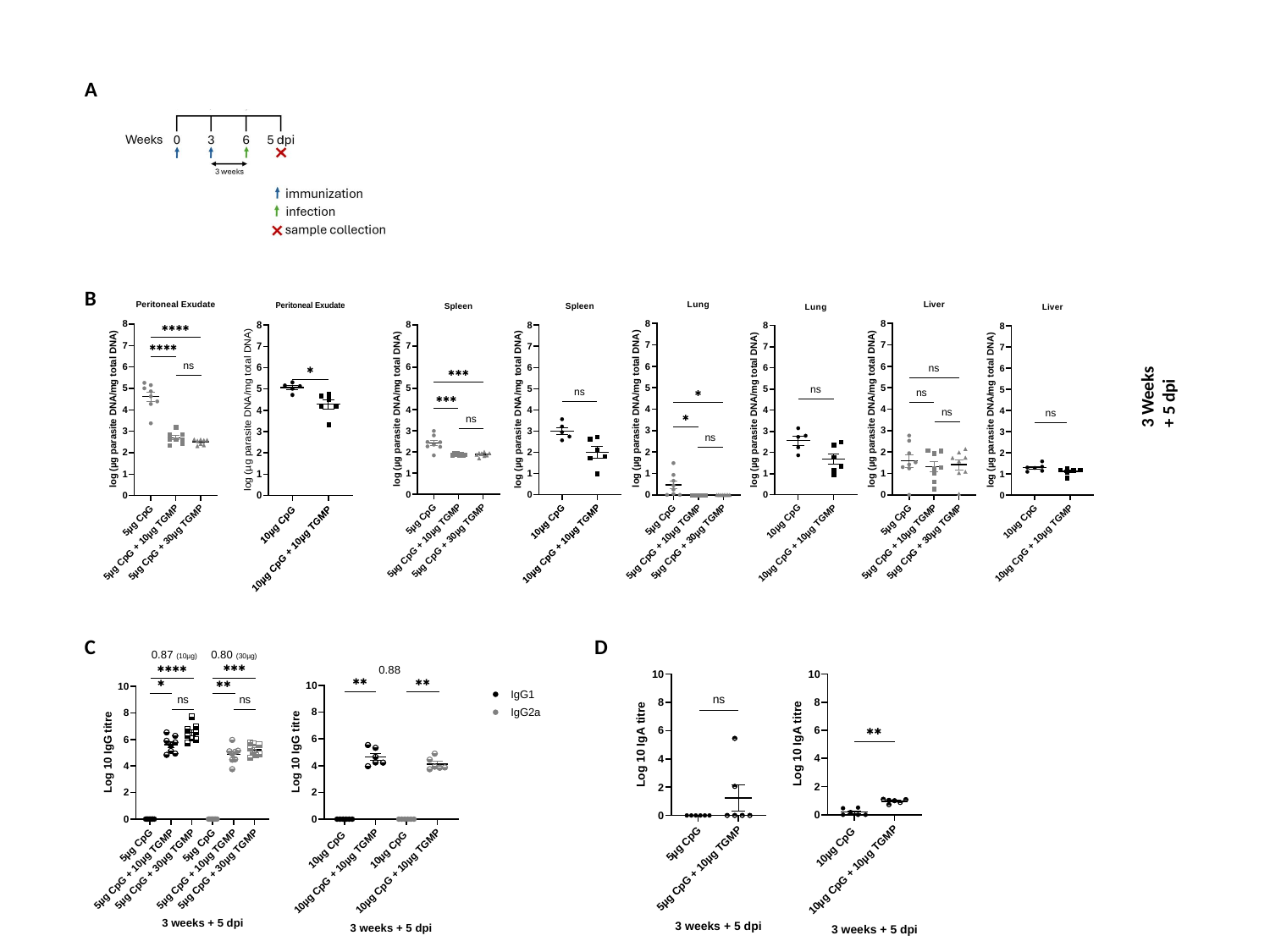

A
B
3 Weeks + 5 dpi
D
C

## Slide 2
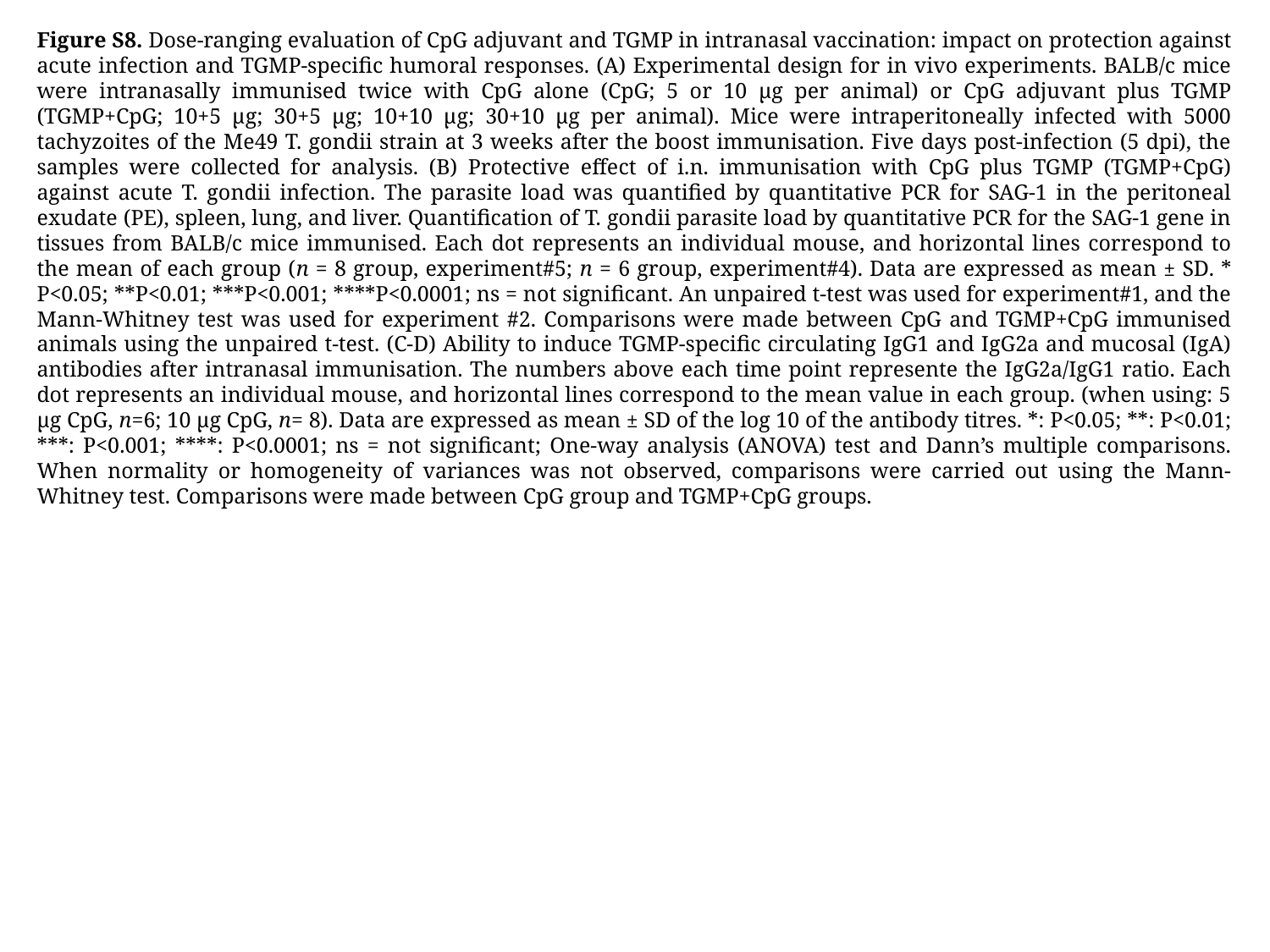

Figure S8. Dose-ranging evaluation of CpG adjuvant and TGMP in intranasal vaccination: impact on protection against acute infection and TGMP-specific humoral responses. (A) Experimental design for in vivo experiments. BALB/c mice were intranasally immunised twice with CpG alone (CpG; 5 or 10 µg per animal) or CpG adjuvant plus TGMP (TGMP+CpG; 10+5 µg; 30+5 µg; 10+10 µg; 30+10 µg per animal). Mice were intraperitoneally infected with 5000 tachyzoites of the Me49 T. gondii strain at 3 weeks after the boost immunisation. Five days post-infection (5 dpi), the samples were collected for analysis. (B) Protective effect of i.n. immunisation with CpG plus TGMP (TGMP+CpG) against acute T. gondii infection. The parasite load was quantified by quantitative PCR for SAG-1 in the peritoneal exudate (PE), spleen, lung, and liver. Quantification of T. gondii parasite load by quantitative PCR for the SAG-1 gene in tissues from BALB/c mice immunised. Each dot represents an individual mouse, and horizontal lines correspond to the mean of each group (n = 8 group, experiment#5; n = 6 group, experiment#4). Data are expressed as mean ± SD. * P<0.05; **P<0.01; ***P<0.001; ****P<0.0001; ns = not significant. An unpaired t-test was used for experiment#1, and the Mann-Whitney test was used for experiment #2. Comparisons were made between CpG and TGMP+CpG immunised animals using the unpaired t-test. (C-D) Ability to induce TGMP-specific circulating IgG1 and IgG2a and mucosal (IgA) antibodies after intranasal immunisation. The numbers above each time point represente the IgG2a/IgG1 ratio. Each dot represents an individual mouse, and horizontal lines correspond to the mean value in each group. (when using: 5 µg CpG, n=6; 10 µg CpG, n= 8). Data are expressed as mean ± SD of the log 10 of the antibody titres. *: P<0.05; **: P<0.01; ***: P<0.001; ****: P<0.0001; ns = not significant; One-way analysis (ANOVA) test and Dann’s multiple comparisons. When normality or homogeneity of variances was not observed, comparisons were carried out using the Mann-Whitney test. Comparisons were made between CpG group and TGMP+CpG groups.
