# Supplementary material for: Protective Effect Against Acute Experimental Toxoplasmosis Conferred by Intranasal Immunisation with Toxoplasma gondii Membrane Proteins Plus CpG Adjuvant
Source: Vaccines (Basel). 2026 Jun 17;14(6):539. doi: 10.3390/vaccines14060539 (PMC13308317; doi:10.3390/vaccines14060539)
Supplement: Supplementary file 1 [file vaccines-14-00539-s001.zip › Table S1.pdf]

Table S1. Animal groups used in this study and corresponding experiments.

Mice groups (total number of animals)	Experiment
10 µg CpG plus 30 µg TGMP (n=37)	#1 (n=8)<br>#2 (n=8)<br>#3 (n=5)<br>#4 (n=6)<br>#9.1 (n=5)<br>#9.2 (n=5)
10 µg CpG plus 10 µg TGMP (n=6)	#4 (n=6)
10 µg CpG (n=37)	#1 (n=8)<br>#2 (n=8)<br>#3 (n=5)<br>#4 (n=6)<br>#9.1 (n=5)<br>#9.2 (n=5)
5 µg CpG plus 30 µg TGMP (n=15)	#5 (n=8)<br>#6 (n=6)
5 µg CpG plus 10 µg TGMP (n=23)	#5 (n=8)<br>#7 (n=9)<br>#8 (n=6)
5 µg CpG (n=23)	#5 (n=8)<br>#6 (n=6)<br>#7 (n=9)<br>#8 (n=6)

Mice were distributed randomly into groups and i. n. immunised twice with an interval of three weeks with: CpG (ODN 1826 VacciGrade, Invivogen, San Diego, CA) at 0.25 µg/mL (5 µg per animal) or 0.5 mg/mL(10 µg per animal) plus TGMP

at 0.5 mg/mL (10 μg per animal) or 1.5 mg/mL (30 μg per animal), all resuspended in saline solution (0.9% NaCl; CpG/TGMP group) or CpG alone at 0.25 μg/mL (5 μg per animal) or 0.5 mg/mL (10 μg per animal) resuspended in saline solution (CpG group, sham-immunized animals).
