# Supplementary material for: Protective Effect Against Acute Experimental Toxoplasmosis Conferred by Intranasal Immunisation with Toxoplasma gondii Membrane Proteins Plus CpG Adjuvant
Source: Vaccines (Basel). 2026 Jun 17;14(6):539. doi: 10.3390/vaccines14060539 (PMC13308317; doi:10.3390/vaccines14060539)
Supplement: Supplementary file 1 [file vaccines-14-00539-s001.zip › Table S2.pdf]

Table S2: Animal weight monitoring after infection

		Animal weight (g)				
Immunisation solution	Intraperitoneal infection	1 dpi	2 dpi	3 dpi	4 dpi	5 dpi
5 µg CpG	21.99±0.21	22.13±0.15	21.85±0.11	22.42±0.19	22.46±0.28	22.58±0.21
5 µg CpG +10 µg TGMP	22.25±0.61	21.84±0.52	21.72±0.48	21.98±0.49	22.21±0.48	22.46±0.49
5 µg CpG +30 µg TGMP	22.91±0.39	23.03±0.35	22.37±0.29	22.42±0.38	22.63±0.39	22.62±0.43

Mice were distributed randomly into groups and intranasally immunised twice with an interval of three weeks with: CpG at 0.25 µg/mL (5 µg per animal) plus TGMP at 0.5 mg/mL (10 µg per animal) or TGMP at 1.5 mg/mL (30 µg per animal), or CpG alone at 0.25 µg/mL (5 µg per animal). Mice were intraperitoneally infected 3 weeks after boost immunisation with 5×10^3 viable tachyzoites of ME49 strain of *T. gondii*. Animals were weighed daily from day 0 to day 5 post-infection. Data represent the mean + SEM of mice analysed individually (n=8 in each group). The present study was performed in accordance with ARRIVE guidelines (https://arriveguidelines.org). dpi: days post-infection.
